# Co-delivery of IL-12/IL-15/IL-18 engineered DC vaccines with anti-IL-10R and nanoconjugated methotrexate in melanoma

**DOI:** 10.3389/fimmu.2026.1773836

**Published:** 2026-03-02

**Authors:** Katarzyna Węgierek-Ciura, Agnieszka Szczygieł, Anna Rudawska, Jagoda Mierzejewska, Joanna Rossowska, Bożena Szermer-Olearnik, Marta Świtalska, Tomasz M Goszczyński, Elżbieta Pajtasz-Piasecka

**Affiliations:** Hirszfeld Institute of Immunology and Experimental Therapy, Polish Academy of Sciences, Wrocław, Poland

**Keywords:** anti-IL-10R antibodies, B16, dendritic cells, interleukin 12, interleukin 15, interleukin 18, melanoma, methotrexate nanoconjugate

## Abstract

**Background:**

In an immunosuppressive microenvironment created by melanoma cells, interleukin (IL)-10 can promote tumor growth and impair the function of antigen-presenting cells, particularly dendritic cells. One of the leading strategies to counteract IL-10’s action is the administration of antibodies against its receptor. The tumor microenvironment can also be modulated by cytokines and/or cellular vaccines such as modified dendritic cells that overproduce IL-12, IL-15/IL-15Rα, and IL-18. These cellular vaccines serve as a source of cytokines and stimulate the immune system by presenting tumor antigens to lymphocytes. Furthermore, the efficacy of the dendritic cell vaccine can be achieved through the nanoconjugate of methotrexate and hydroxyethyl starch (HES-MTX), which reduces the activity of IL-10 and eliminates immunosuppressive cells.

**Methods:**

Two experiments were conducted: one focusing on immunotherapy and the other on chemoimmunotherapy. The immunotherapy involved two administrations of cellular vaccines, preceded by anti-IL-10R antibody treatment. The chemoimmunotherapy additionally included a single administration of the HES-MTX nanoconjugate. The effectiveness of both therapies was evaluated through tumor growth inhibition measurements and analysis of lymphoid and myeloid cell populations in tumor tissues. Additionally, subpopulations of restimulated splenocytes were analyzed, and the production levels of interferon gamma (IFN-γ), IL-10, and IL-4 were evaluated.

**Results:**

Modified dendritic cells, which carry proinflammatory cytokines, were used in immuno- and chemoimmunotherapeutic experiments. The developed therapies effectively inhibited tumor growth, but the rate of tumor growth depended on the type of vaccine used. Incorporating the nanoconjugate prior to immunological treatment primarily reduced the population of suppressor cells. The most effective treatment was observed in two cases: as a result of immunotherapy including the use of a two-component vaccine DC/IL-12/TAg + DC/IL-18/TAg (TGI 62.3%) or after administration of HES-MTX nanoconjugate followed by immunotherapy with three-component vaccine - DC/IL-12/TAg + DC/IL-15/IL-15Rα/TAg + DC/IL-18/TAg (TGI 59.1%).

## 1 Introduction

Melanoma is a rapidly growing tumor that creates a hostile environment, in which interleukin (IL)-10 plays a key role, engaging the tumor microenvironment (TME) and supporting its growth by stimulating cell proliferation, angiogenesis, and activating immunosuppressive mechanisms (1). Although interleukin 10 may be associated with increased infiltration of CD8^+^ cells into the TME and increased interferon gamma (IFN-γ) release during the initial phase of tumor growth (2), in general, it is an immunosuppressive factor. It inhibits antigen presentation by downregulation of MHC class II expression in antigen-presenting cells (APCs), their differentiation, maturation, and migration to secondary lymphoid organs, and is involved in the suppression of IL-12 secretion by dendritic cells (DCs). Therefore, several methods to block IL-10 activity have been developed, e.g. by directly promoting the activation of intratumoral CD8^+^ cells (3, 4) or intratumoral application of a lentiviral vector encoding IL-10-silencing short hairpin RNA (shRNA) (5, 6), as well as by blocking IL-10R to enhance myeloid cell function (7).

The melanoma cancer cells influence the development of an immunosuppressive microenvironment, contributing to the low number of cytotoxic T and natural killer (NK) cells and immunosuppressors such as myeloid-derived suppressor cells (MDSCs), tumor-associated macrophages (TAMs), and regulatory T cells (Tregs). It should be underlined that MDSCs, which are responsible for IL-10 production, can transform into TAMs under hypoxic conditions, increasing the size of this population (8, 9). The differentiation of TAM activity is related to the level of MHC class II expression on their surface and the production of antagonistic cytokines such as IL-12 and IL-10, affecting the characteristics of infiltrating lymphocytes. On the other hand, under the influence of MDSCs, the conversion of CD4^+^ cells into regulatory T cells can occur. Tregs suppress anti-tumor immune responses primarily by promoting an immunosuppressive microenvironment through cytokine secretion, including IL-10 and transforming growth factor-β (10, 11). Clinic observations confirm that an increased incidence of Tregs is associated with a worse prognosis in patients with cancer. For this reason, restoring immune balance and strengthening immune function are essential aspects of immunotherapy. It can be achieved through the use of proinflammatory cytokines such as IL-12, IL-15, and IL-18, which have the ability to affect multiple immune system cells.

Interleukin 12 can regulate inflammation using effector mechanisms of both innate and acquired immunity, especially those mediated by IFN-γ, such as the differentiation of T helper type 1 (Th1) cells (12, 13). The influence of IL-12 on the antitumor response has been observed in preclinical models, but its dose-related toxicity has limited its clinical use (14). Nevertheless, the clinical use of IL-12, especially in combination with other forms of immunotherapy and other therapeutic drugs, is still under investigation. The next cytokine, IL-15, triggers the activation, proliferation, survival, and cytotoxicity of CD8^+^ cells and NK cells. It is also critical for the functional maturation of both macrophages and dendritic cells. Studies in different models with multiple murine immunotherapies have revealed that IL-15 might be more effective than IL-2 (15, 16). However, IL-15 administered as monotherapy has proved ineffective, necessitating combination with other anticancer agents (17). Therefore, it can be used with interleukin 18, which is particularly produced by macrophages and DCs. Interleukin 18 has been described as functionally similar to, but more potent than, IL-12 in activating T lymphocytes and NK cells to produce IFN-γ. *In vivo* studies have confirmed the importance of IL-18 and IL-12 cooperating in IFN-γ production and the polarization of the Th1 response (18, 19). Its contribution includes promoting functional development, T and NK cell maturation, enhancing cytotoxicity, and increasing IL-4 and IL-13 production. Some clinical trials have investigated recombinant human IL-18 (SB-485232) in treating melanoma, lymphoma, and ovarian cancers [NCT00500058, NCT01768338, NCT02277392].

Nevertheless, the use of soluble forms of cytokines can be associated with severe side effects, so a safer alternative may be the use of dendritic cells as carriers of these cytokines. DCs modified to overexpress cytokines are able to produce them for a longer period of time but in smaller quantities. These cells, with unique antigen presentation properties, loaded with tumor products in both mouse and human systems, are recognized as an attractive tool in anticancer therapy (20). DCs genetically modified to produce cytokines should affect a decrease in the Treg cell population size or a shift in the balance between Tregs and T-effector cells, leading to the restoration of the immune response (21). Moreover, appropriately matured DCs are reported to generate specific immunity against defined tumor targets. Supplemented by overproduced proinflammatory cytokines, these would stimulate other immune cells to contribute to tumor tissue reduction.

The effect of immunotherapy can be enhanced by using a chemotherapy agent at an immunomodulating dose. One of the promising agent is methotrexate nanoconjugate (HES-MTX). The nanoconjugate has a longer plasma half-life (approximately 65.5 ± 5 hours in plasma) than the free form of MTX (approximately 6–15 hours in plasma), and improved drug biodistribution in the body (22, 23). Such modification aids the nanoconjugate in entering cells through folate receptors (FRs), especially FRα, which is overexpressed on cancer cells. This allows the nanoconjugate to extend the half-life of the cytostatic attached to the carrier, increasing the likelihood of the drug reaching tumor tissue instead of healthy tissue (23).

In our recent studies in the MC38 colon cancer model, we demonstrated the immunomodulatory potential of the HES-MTX nanoconjugate, which manifested itself in an increase of immune cell influx and the elimination of cells with suppressor activity in the tumor tissue, followed by the induction of a specific anti-tumor immune response (24). Further studies revealed that this was a beneficial factor supporting the therapeutic activity of dendritic cell-based vaccines, especially when they were additionally supported by abolishing the negative effect of IL-10 on their activity, e.g., by downregulating IL-10R expression in DCs (25) or systemic use of anti-IL-10R antibody (26). Therefore, considering the immunomodulatory potential of HES-MTX in B16 F0 tumor model, and due to the urgent need to develop new immunotherapeutic regimens for this type of cancer, we decided to combine the immunomodulatory dose of HES-MTX nanoconjugate with immunotherapy consisting of cellular vaccines with DCs genetically modified to overproduce the proinflammatory cytokines: IL-12, IL-15 and IL-18. Moreover, to reduce the adverse effect of the TME-derived IL-10 on the immune response, this combined therapy was extended to include systemic administration of anti-IL-10R antibodies prior to every cellular vaccine injection. We examined the immune response against B16 F0 melanoma when the immunotherapy was applied alone, but also when it was preceded by the use of a HES-MTX. The developed immunotherapy was effective in inhibiting tumor growth; however, the rate of tumor growth depended on the type of vaccine used. Significant tumor growth inhibition was observed after the administration of the two-component vaccine, DC/IL-12/TAg + DC/IL-18/TAg. In this group, a considerable influx of CD45^+^ cells into the tumor tissue, along with the strong stimulation of restimulated splenocytes were observed. Following chemoimmunotherapy the highest percentage of CD45^+^ cells infiltrating the tumor tissue was observed in groups of mice that received the two-component vaccine DC/IL-15/IL-15Rα/TAg + DC/IL-18/TAg or the three-component vaccine. Incorporating nanoconjugate prior to immune-treatment reduced among CD45^+^ cells the percentage of tumor-associated macrophages, CD8^+^ and CD4^+^ cells, and regulatory T cells among CD4^+^ cells. However, the percentage of CD107a^+^ cells within the CD4^+^ cell population increased among restimulated splenocytes, unlike a slight decrease in the percentage of CD107a^+^ cells among CD8^+^ and NK cells compared to those undergoing immunotherapy alone.

The effect of therapies depended on the combination of cytokines rather than their levels, but the strength of this phenomenon appeared to be related to the type of cytokine combination. These results imply the need for a deeper consideration how to create combinations of individual components and to rationally analyze their emerging effects.

## 2 Materials and methods

### 2.1 Animals

Female C57BL/6 mice were obtained from the Center for Experimental Medicine of the Medical University of Bialystok (Białystok, Poland). Mice were housed in a standard light/dark cycle room with a constant temperature of 22 ± 2˚C, air humidity of 55 ± 10%, and unlimited access to food and water. All experimental procedures were by the guidelines set out in the EU Directive 2010/63/EU on animal testing and were approved by the Local Ethics Committee for Animal Experiments in Wrocław (permit number: 009/2021; 005/2022). Following the experiment, the mice were humanely sacrificed by cervical dislocation.

### 2.2 Tumor cell lines and lysate preparation

The non-metastatic murine melanoma B16 F0 cell line (ECACC 92101204) was cultured in high glucose Dulbecco’s Modified Eagle Medium (DMEM, ATCC) supplemented with 100 U/ml penicillin, 100 mg/ml streptomycin and 10% heat-inactivated fetal bovine serum (FBS; Sigma−Aldrich). The cultures were maintained under typical conditions, which involving 5% CO_2_ and a temperature of 37 °C.

To prepare a lysate from tumor cells, the B16 F0 melanoma cells were gathered and suspended at a density of 5 × 10^6^ cells/ml in RPMI-1640 (Gibco) supplemented with 10% FBS (Sigma-Aldrich). All cells underwent five freeze cycles in liquid nitrogen followed by rapid thawing at 37 °C. Subsequently, the cell suspension was sonicated for 90 minutes. The resulting B16 F0 tumor antigens (TAg) were utilized for stimulating dendritic cells.

### 2.3 Lentiviral vector production

Lentiviral vectors (LVs) were generated using a third-generation lentiviral system, which included auxiliary plasmids: pMDLg/pRRE, pRSV Rev, and pMD2.G (these plasmids were generously provided by Didier Trono, Addgene plasmids #12251, 12253, 12259), along with the expression vector. Various cytokine genes were encoded in the expression plasmids: *il12*, *il15/il15ra*, or *il18*. For the *il15*-carrying vector, the cytokine’s gene sequence was preceded by a signal sequence to facilitate its extracellular release. Furtheremore an alpha subunit of the *il15* receptor gene sequence was incorporated to slow down intracellular protein degradation. The control vector (pLVctrl) was designed to assess the effect of lentiviral transduction on dendritic cells. All expression vectors were sourced from VectorBuilder.

Lentiviral vectors were obtained through co-transfection of Lenti-X 293T cells (ClonTech) with the aforementioned plasmids. The Lenti-X 293T cells were cultured with plasmids for 48 hours. The supernatant, enriched with lentiviral vectors, was harvested and subjected to concentration via precipitation utilizing PEG 6000 (Sigma-Aldrich). The pellet, containing the lentiviral vectors, was resuspended in PBS and preserved at -80 °C. The protocol for lentiviral vector production was delineated in a prior publication (27). To determine the lentiviral vector’s titer, a serial dilution method was employed, and flow cytometry analysis.

### 2.4 Preparation of DC vaccines

Dendritic cells were derived from bone marrow extracted from the femurs and tibias of healthy female C57BL/6 mice, following the methodology outlined in our prior publication (28). These cells, referred to as DCs hereafter, were cultured in RPMI-1640 (Gibco) with 10% FBS (Sigma-Aldrich). The culture was supplemented with recombinant murine (rm)GM-CSF (ImmunoTools, 40 ng/ml) and rmIL-4 (ImmunoTools, 10 ng/ml).

Following 7 days of cultivation, immature DCs were subjected to transduction with LVs, using the assumption of 4 viral infectious particles per 1 dendritic cell. This transduction was carried out in the presence of 8 µg/ml polybrene (Sigma-Aldrich). DCs were transduced by lentiviral vectors carring *il12*, *il15/il15ra*, *il18* genes or by control vector and will be referred to as modified dendritic cells. Four hours after transduction the DCs were stimulated with tumor antigen lysates (TAg, 10% v/v). Mature dendritic cells, which were acquired on the eighth day of DC culture, were harvested and employed for examining *in vitro* DCs characteristics. Additionally, these mature dendritic cells were utilized as cellular vaccines in subsequent *in vivo* experiments.

### 2.5 Analysis of cytokine production

Cytokine production was assessed by utilizing commercially accessible ELISA kits (IL-10, IL-4 (BD Biosciences); IL-12, IL-15/IL-15Rα, IL-18, IFN-γ (eBioscience)), following the guidelines provided by manufacturers.

### 2.6 Phenotype characteristics of DC vaccines

To assess the impact of LV transduction on DC differentiation levels, we conducted phenotype characterization on the 10^th^ day of DC culture through flow cytometry. For this purpose, cells were labeled using a cocktail of monoclonal antibodies conjugated with fluorochromes: anti-CD11c Brilliant Violet 650 (clone N418), CD80 PerCP-Cy5.5 (clone 16-10A1), CD86 PE-Cy7 (clone GL-1), MHC II APC-Fire 750 (clone M5/114.15.2) – all sourced from BioLegend, and CD40 Brilliant Violet 605 (clone 3/23) from BD Biosciences. To exclude non-viable cells, DAPI dye (Invitrogen) was used before analysis. The flow cytometric analysis was executed using the LSRFortessa flow cytometer with Diva software (BD Biosciences).

### 2.7 Primary stimulation of splenocytes by modified DCs

The efficacy of the modified DCs in initiating a primary antigen-specific immune response was appraised through a 5-day co-culture involving mature DCs (0.18 × 10^6^ cells) and naive spleen cells (1.8 × 10^6^ cells). This co-culture was established on the 8^th^ day of DC cultivation and was conducted in the presence of recombinant human (rh)IL-2 (200 U/ml, ImmunoTools).

### 2.8 CD107a degranulation assay

Restimulated or primary stimulated spleen cells obtained from 5-day co-culture were subjected to a 2-hour incubation with B16 F0 cells. This incubation occurred in the presence of monoclonal anti-CD107a antibodies conjugated with APC (clone 1D4B, BioLegend), ionomycin (1 µg/ml, Sigma-Aldrich), phorbol-12-myristate-13-acetate (50 ng/ml, Sigma-Aldrich), and rhIL-2 (200 U/ml). Post-incubation, the cells were gathered and stained with anti-CD45 Brilliant Violet 605 (clone 30-F11), anti-CD4 FITC (clone RM4-5), anti-CD8a APC-Fire (clone 53-6.7), and anti-NK1.1 PE-Dazzle 594 (clone PK136) – all obtained from BioLegend. DAPI dye was utilized to exclude non-viable cells. The flow cytometry analysis was conducted employing the LSRFortessa flow cytometer with Diva software (BD Biosciences).

### 2.9 Spleen cell cytotoxicity assay

After five days of primary stimulation, cells were harvested and the cytotoxic activity of effector splenocytes against DiO^+^ labeled (Molecular Probes) B16 F0 melanoma cells was analyzed, according to the previously described procedure (29). The dead target cells were distinguished with propidium iodide (PI, Thermo Fisher Scientific) solution and the percentage of DiO^+^PI^+^ B16 F0 cells was determined.

### 2.10 Therapeutic treatment schedule

Female C57BL/6 mice aged eight to ten weeks were used in experiment. B16 F0 melanoma cells (3.0 × 10^4^ cells/0.1 ml Matrigel/mouse) were subcutaneously injected into the right flank of the mice. Once palpable tumor nodules were observed, the mice were divided into experimental groups based on tumor volume. Eleven groups were formed for immunotherapy, and twelve groups were formed for chemoimmunotherapy. In both experiments, cellular vaccines were administered peritumorally (p.t.) on the 11^th^ and 16^th^ days (2 × 10^6^ cells/0.2 ml NaCl 0.9%/mouse/injection). The anti-IL-10R antibodies (BioXCell) at a dose of 250 µg/0.2 ml/mouse/injection were administered intraperitoneally (i.p.) the day before injection of the cellular vaccines (10^th^ and 15^th^ day of the experiment). The therapeutic schedule of chemoimmunotherapy was started on the 8^th^ day of the experiment with intravenous (i.v.) HES-MTX nanoconjugate injection into the tail vein (at a dose of 20 mg/kg body weight). Detailed information about the HES-MTX preparation can be found in our previous papers (24). The cellular vaccines used in both experiments included three types: non-transduced DC/TAg, DC transduced with a control vector (DC/Vctrl/TAg), and DCs engineered to overproduce cytokines. Each mouse received 2 × 10^6^ modified dendritic cells per injection, regardless of whether the cells overproduced a single cytokine or a combination of two or three cytokines. Cellular vaccines named DC/IL-12/TAg, DC/IL-15/IL-15Rα/TAg and DC/IL-18/TAg consisted of 2 × 10^6^ cells of appropriate transductants, DC/IL-12/TAg + DC/IL-15/IL-15Rα/TAg, DC/IL-12/TAg + DC/IL-18/TAg and DC/IL-15/IL-15Rα/TAg + DC/IL-18/TAg consisted of 1 × 10^6^ cells of each listed transductants, while DC/IL-12/TAg + DC/IL-15/IL-15Rα/TAg + DC/IL-18/TAg was a mixture of three different transductants in the number of 0.667 × 10^6^ cells each.

### 2.11 Kinetics of tumor growth in mice

During the *in vivo* experiment, tumor growth was regularly monitored and measured using an electronic caliper. The volume of tumors was calculated according to the formula: a2×b2, where *a* is the largest and *b* is the smallest diameter of the tumor. To analyze tumor growth kinetics, the nonlinear least squares regression fits of the Gompertz function were utilized. Therapeutic efficacy was determined by calculating the tumor growth inhibition (TGI) value as follows: $TGI\left(\%\right)=100-\left(TV_{t}/TV_{nt}\times 100\right)$, where $TV_{t}$ refers to a mean tumor volume in the treated group and $TV_{nt}$ – mean tumor volume in the non-treated (nt) group. Three days after the final injection of the DC-based vaccine (19^th^ day of therapy), spleens and tumor nodules from B16-tumor-bearing mice were dissected, homogenized, and preserved in liquid nitrogen for subsequent *ex vivo* analyses.

### 2.12 Analysis of myeloid and lymphoid cells in B16 tumor nodules after therapy

The cell suspension obtained from tumor tissue was thawed and labeled with monoclonal antibodies conjugated to fluorochromes. The following antibodies were used to determine the population of myeloid and lymphoid cells infiltrating the tumor tissue: anti−CD45 Brilliant Violet 605 (clone 30-F11), anti−CD3 PE−CF594 (clone 145-2C11), anti−CD19 PE−CF594 (clone 1D3), anti−NK1.1 PE−Dazzle 594 (clone PK136) (all from BD Biosciences), anti−CD11b PerCP−Cy5.5 (clone M1/70), anti−CD11c Brilliant Violet 650 (clone N418), anti−F4/80 Alexa Fluor 700 (clone BM8), anti−Ly6C PE (clone HK1.4), anti−MHC II FITC (clone M5/114.15.2) (all from BioLegend) for myeloid cell identification, and anti−CD45 Brilliant Violet 605 (clone 30-F11), anti−CD3 Brilliant Violet 650 (clone 17A2), anti−CD4 FITC (clone RM4-5), anti−CD8 APC/Fire 750 (clone 53-6.7), anti-CD19 Alexa Fluor 700 (clone 6D5), anti−CD25 PE (clone PC61) (all from BioLegend) for lymphocyte identification. Then, cells were fixed using the Foxp3/Transcription Factor Staining Buffer Set (eBioscience). Cells stained with lymphocyte cocktail were additionally incubated with anti−FoxP3 APC (clone FJK-16s) (eBioscience) monoclonal antibodies. The flow cytometry analysis was performed using the LSR Fortessa flow cytometer with Diva software (BD Biosciences).

### 2.13 Analysis of the systemic anti-tumor immune response of splenocytes after therapy

Spleen single-cell suspensions (2.0 × 10^6^ cells) were thawed and cocultured with mitomycin C-treated B16 F0 cells (0.1 × 10^6^ cells) in the presence of rhIL−2 (200 U/ml). After 5 days of restimulation, cells were harvested and the degranulation assay was used according to the previously described procedure (section: Primary stimulation of splenic cells by transduced dendritic cells). Supernatants were collected and stored at 4 °C until ELISA was performed.

### 2.14 Statistical analyses

Statistical analysis was performed using the GraphPad Prism 9 software (GraphPad Software, Inc.). The analysis of tumor tissue-infiltrating cells and spleen cells was performed from 3–6 mice (nt - 6, Ab - 5, I - 4, II - 4, III - 4, IV - 5, V - 6, VI - 5, VII - 5, VIII - 3, IX - 5) in the case of immunotherapy and 5-9 (nt - 8, H-M - 7, Ab - 6, I - 8, II - 9, III - 8, IV - 6, V - 6, VI - 6, VII - 5, VIII - 6, IX - 7) in the case of chemoimmunotherapy. The normality of the data distribution was assessed using the Shapiro-Wilk test (**Supplementary Tables 1**-**4**). For data conforming to a Gaussian distribution (p > 0.05) with uniform standard deviation (SD) values, statistical significance was determined using the parametric one−way ANOVA, followed by Tukey’s multiple comparison post−hoc test. In cases where data aligned with a Gaussian distribution but exhibited unequal SD values, we employed the Brown−Forsythe and Welch ANOVA tests, followed by Dunnett’s T3 multiple comparisons post−hoc test. Data that didn’t adhere to a Gaussian distribution (p ≤ 0.05) underwent analysis using the nonparametric Kruskal−Wallis test, followed by Dunn’s multiple comparison post−hoc test. Number of mice considered for evaluating tumor growth kinetics in immunotherapy: nt - 6, Ab - 7, I - 4, II - 6, III - 5, IV - 6, V - 7, VI - 7, VII - 5, VIII - 3, IX - 7 and chemoimmunotherapy: nt - 8, H-M - 8, Ab - 8, I - 8, II - 10, III - 6, IV - 7, V - 8, VI - 8, VII - 6, VIII - 7, IX - 7. The statistical significance concerning tumor growth kinetics was assessed using the two−way ANOVA, followed by Tukey’s multiple comparisons post−hoc test. Specific details regarding the type of statistical analysis employed can be found in the figure captions. Graphs portray all statistically significant differences using symbols outlined in **Table 1**.

**Table 1:** Statistical significance markings on graphs.

P value	nt	HES-MTX (H-M)	DC/Vctrl/TAg	other
< 0.0001	****	####	xxxx	**** above the line
0.0001 to 0.001	***	###	xxx	*** above the line
0.001 to 0.01	**	##	xx	** above the line
0.01 to 0.05	*	#	x	* above the line
≥ 0.05	no check mark	no check mark	no check mark	no check mark

## 3 Results

### 3.1 Characteristics of genetically modified DC-based vaccines

The study began with the characterization of the obtained cellular vaccines and the evaluation of their ability to trigger immune responses against B16 F0 melanoma. To achieve this, bone marrow-derived dendritic cells were transduced with lentiviral vectors carrying the genes *il12*, *il15*/*il15Rα*, or *il18*. Additionally, these genetically modified dendritic cells were stimulated with B16 F0 tumor cell lysate (tumor antigens, TAg). Control groups included DCs transduced with a control vector or non-transduced DCs stimulated with TAg (referred to as DC/Vctrl/TAg or DC/TAg, respectively). The vaccine preparation scheme has been detailed in our previous publications (26).

We tested the effectiveness of vaccines consisting of DCs producing one cytokine – one-component vaccine (DC/IL-12/TAg, DC/IL-15/IL-15Rα/TAg, DC/IL-18/TAg), a 1:1 mixture of two types of DCs – two-component vaccine (DC/IL-12/TAg + DC/IL-15/IL-15Rα/TAg; DC/IL-12/TAg + IL-18/TAg and DC/IL-15/IL-15Rα/TAg + DC/IL-18/TAg), or a 1:1:1 mixture of three types of DCs – three-component vaccine. To evaluate DC activity in *in vitro* and ex vivo assays, we ensured that the same number of cells was used for each well or single injection, regardless of the vaccine composition.

**Figure 1:**
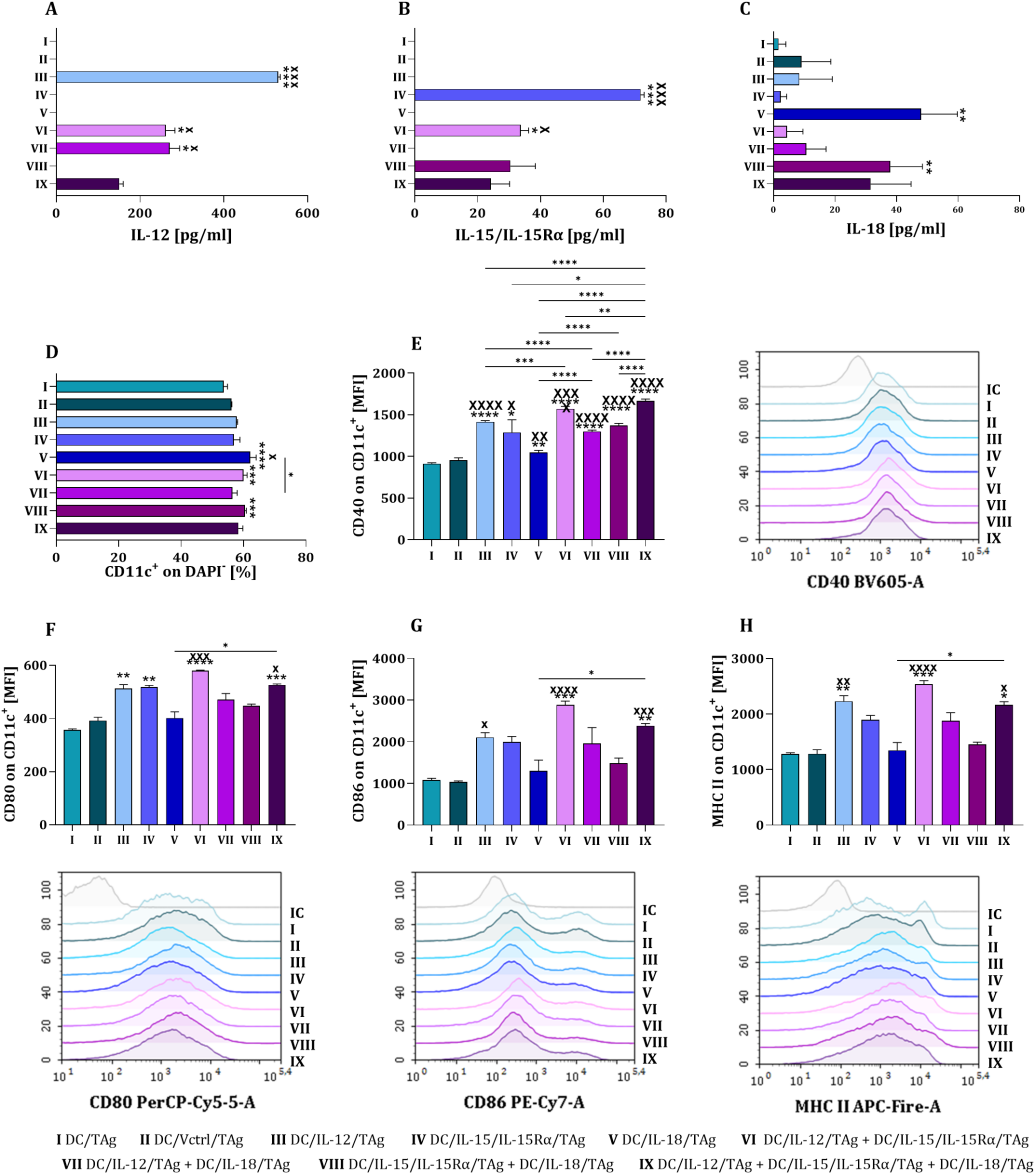
Efficiency of lentiviral transduction with *il12, il15* and *il15Rα* or *il18* genes and the level of differentiation of bone marrow-derived dendritic cells (DCs) stimulated with B16 F0 tumor antigens (TAg). Concentration of overexpressed cytokines IL-12 **(A)**, IL-15 **(B)**, IL-18 **(C)** in supernatants collected after 48 hours of DCs cultures measured using ELISA. Percentage of CD11c^+^ cells on the 10^th^ day of DCs cultured **(D)**. Expression of CD40 **(E)**, CD80 **(F)**, CD86 **(G)** and MHC II **(H)** molecules on the surface of CD11c^+^ cells (MFI) (bar plots and representative overlay histograms). Results are presented as mean+SD calculated for 5–6 samples per group. Differences between groups were estimated using the non-parametric Kruskal-Wallis test followed by Dunn’s multiple comparisons *post-hoc* test **(A–D, F–H)** or the parametric Brown-Forsythe and Welch ANOVA test followed by Dunnett’s T3 multiple comparisons *post-hoc* test **(E)**. The asterisks (*) presented in the graphs indicate statistically significant differences between the given groups and the DC/TAg control cells; crosses (X) indicate a statistically significant difference between the given group and the DC/Vctrl/TAg control cells, asterisks (*) under the line indicate statistically significant differences between the given groups – (*/^x^p<0.05; **/^xx^p<0.01; ***/^xxx^p<0.001; ****/^xxxx^p<0.0001). IC – *isotype control*, MFI *– mean fluorescence intensity*.

For this purpose, the dendritic cells were cultured for 48 hours, after which their ability to secrete cytokines was evaluated (**Figures 1A–C**). The highest concentrations of IL-12 (**Figure 1A**) and IL-15 (**Figure 1B**) were observed in cultures with a single type of dendritic cells. In two-component and three-component cell cultures, a proportional decrease in the concentrations of these cytokines was noted, corresponding to the number of cells of a particular cell type. However, all types of dendritic cells were capable of IL-18 secretion (**Figure 1C**). The highest concentration of IL-18 was recorded in the DC/IL-18/TAg group which confirms the effectiveness of the introduced genetic modification. At the same time, slightly lower levels were found in the mixed cultures containing DC/IL-15/IL-15Rα/TAg + DC/IL-18/TAg, as well as in the three-component mixed culture. Notably, the concentration of IL-18 in the mixed two-component culture containing DC/IL-12/TAg + IL-18/TAg was low.

We analyzed whether interleukins overexpression affects phenotypic and functional changes in dendritic cells on the 10^th^ day of culture. We found that the presence of cytokines in the culture resulted in a statistically significant increase in the percentage of DCs (CD11c^+^DAPI^-^) compared to control cells. It was observed when IL-18 was present in the culture alone or when IL-15/IL-15Rα was combined with other factors (IL-12/TAg + IL-15/IL-15Rα/TAg or IL-15/IL-15Rα/TAg + IL-18/TAg) (**Figure 1D**). Additionally, the overproduction of these cytokines led to greater variation in the expression levels of costimulatory molecules CD40, CD80, CD86, as well as MHC II molecules (**Figures 1E–H**). The most substantial effect was observed in the presence of IL-12, while the smallest was in the culture of IL-18. Expression of CD40 on the DC cell surface, estimated by mean fluorescence intensity (MFI), was the highest when all three cytokines appeared together in the mixed culture (DC/IL-12/TAg + DC/IL-15/IL-15Rα/TAg + DC/IL-18/TAg). In contrast, the highest expression of CD80, CD86, and MHC II molecules occurred in a two-component mixed culture: DC/IL-12/TAg + DC/IL-15/IL-15Rα/TAg.

Based on the preliminary characterization of DCs genetically modified with the *il12, il15* and *il15Rα* or *il18* genes, we found that developed cells can be carriers of these cytokines and constitute their significant source in the tumor microenvironment. Moreover, these vaccine cells had a high potential to increase immune cell activation due to their higher maturity associated with the expression of costimulatory molecules.

**Figure 2:**
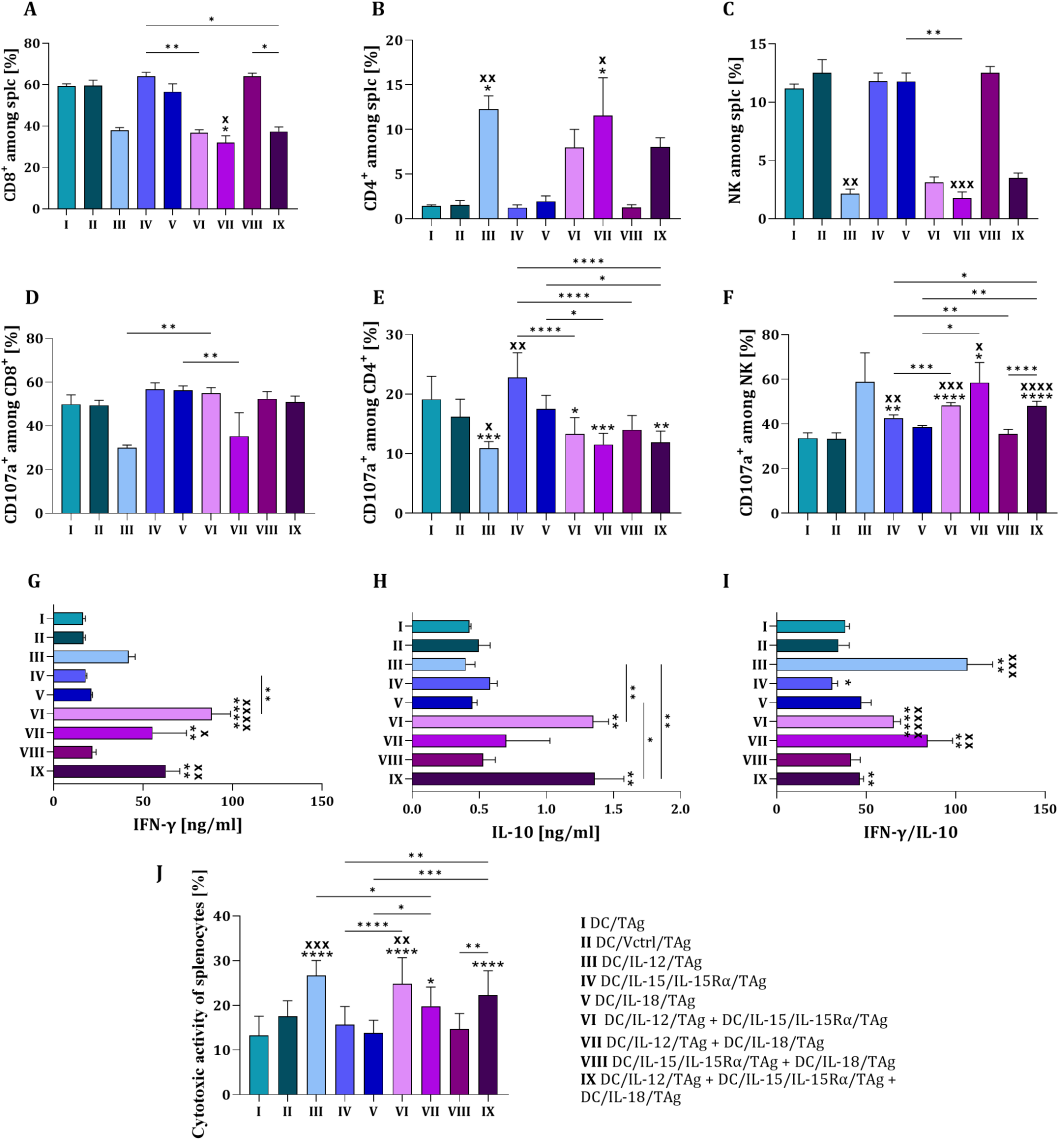
The ability of DCs genetically modified to produce IL-12, IL-15/Il-15Rα or IL-18 and stimulated with B16 F0 tumor antigens to prime splenocytes. Percentage of CD8^+^ cells **(A)**, CD4^+^ cells **(B)** and NK cells **(C)** among splenocytes obtained after 5-day coculture with DCs. Percentage of effector cells (CD107a^+^) among CD8^+^**(D)**, CD4^+^**(E)** and NK cells **(F)** after 2-hour incubation with B16 F0 cells. Concentration of IFN-γ **(G)** and IL-10 **(H)** in supernatants collected after 5 days of cocultured DCs and spleen cells. Ratio of IFN-γ and IL-10 concentration **(I)**. Cytotoxic activity of splenocytes presented as a percentage of dead B16 F0 tumor cells after 4-hour incubation with effector cells in a ratio of 10:1 (effector:target) **(J)**. Results are presented as mean+SD calculated for 6–12 samples per group. Differences between groups were estimated using the non-parametric Kruskal-Wallis test followed by Dunn’s multiple comparisons *post-hoc* test **(A–D, G–I)**, the parametric one-way ANOVA followed by Tukey’s multiple comparisons *post-hoc* test **(E, J)** or the parametric Brown-Forsythe and Welch ANOVA test followed by Dunnett’s T3 multiple comparisons *post-hoc* test **(F)**. The asterisks (*) presented in the graphs indicate statistically significant differences between the given groups and the DC/TAg control cells; crosses (X) indicate a statistically significant difference between the given group and the DC/Vctrl/TAg control cells, asterisks (*) under the line indicate statistically significant differences between the given groups – (*/^x^p<0.05; **/^xx^p<0.01; ***/^xxx^p<0.001; ****/^xxxx^p<0.0001).

To evaluate the ability of different dendritic cell types to induce a primary stimulation of naïve T cell activity, we conducted a 5-day co-culture with splenocytes (**Figure 2**). We analyzed changes in CD8^+^, CD4^+^ and NK splenocyte subpopulations. The presence of IL-12 produced by transduced DCs during the primary stimulation of spleen cells resulted in noticeable changes in specific lymphoid cell types. Whether IL-12 was administered alone or in combination with IL-15 or IL-18, we observed a decrease in the percentage of CD8^+^ cells and NK cells, while the population of CD4^+^ T cells increased (**Figures 2A–C**). The percentages of CD107a on the CD8^+^ and CD4^+^ cells were downregulated (**Figures 2D, E**), meanwhile, on the NK cells, increased regardless of whether IL-12 was produced by DCs alone (at the highest concentration) or in connection with other cytokines (**Figure 2F**).

It is noteworthy that the high percentage of active CD8^+^CD107a^+^ cells in mixed cultures lacking IL-12 or containing its low level (group IX) may be related to a slower rate of response compared to conditions with high IL-12 concentrations. Moreover, differences in the size of the CD4^+^CD107a^+^ cell subpopulation were more closely associated with the overproduction of IL-15/IL-15Rα. The presence of IL-12 (groups III, VI, VII, and IX) appeared to be necessary for a significant increase in the NK cell subpopulation.

We also examined the ability of stimulated splenocytes to produce IFN-γ and IL-10. Production of IFN-γ (**Figure 2G**) was strongly increased when cell stimulation was supported not only by IL-12, but also by IL-15 or IL-18 regardless of IL-12 concentration. This finding confirms previous results showing that a mixture of dendritic cells capable of producing different pro-inflammatory cytokines can be a better stimulator than a one-component vaccine.

A similar pattern was observed for the production of IL-10 (**Figure 2H**), which was enhanced when splenocytes were stimulated with the combination of DC/IL-12/TAg + DC/IL-15/IL-15Rα/TAg (group VI) or DC/IL-12/TAg + DC/IL-15/IL-15Rα/TAg + DC/IL-18/TAg (group IX). By analyzing the ratio of IFN-γ to IL-10 (**Figure 2I**), we indicated that IL-12 produced by transduced DCs significantly affected the primary stimulation of spleen cells. We also confirmed that its combination with IL-15 and/or IL-18 was substantially more effective in generating a strong response.

The cytotoxicity of splenocytes against B16 F0 melanoma cells confirmed the significant role of IL-12, which was overproduced by DC/IL-12/TAg. This effect was also noted at its lower concentrations when the cytokine was supported by DC/IL-15/IL-15Rα/TAg and DC/IL-18/TAg (**Figure 2J**).

Therefore, dendritic cells transduced with cytokine genes and stimulated with B16 F0 tumor antigens proved effective in inducing specific cellular responses under ex vivo conditions. By primarily overproducing IL-12, and especially in the presence of secreted IL-15 and IL-18, these cells could activate CD4^+^, CD8^+^, and NK cells, leading to high production levels of IFN-γ and IL-10. Additionally, based on the IFN-γ/IL-10 ratio, we propose that our cellular vaccine preparation method can effectively generate potent inducers of the anticancer immune response.

All types of cell vaccines characterized in *in vitro* experiments were used in the *in vivo* model.

### 3.2 The influence of cytokine-producing DC-based vaccines on the inhibition of tumor growth

One of the most critical factors influencing the effectiveness of therapy is the number of injected cells. In our previous studies, we confirmed that a dose of 2 million vaccine cells could induce an effective immune response, leading to significant inhibition of tumor growth (19, 26). Therefore, we decided to administer 2 million cells at a time, regardless of the combination of cytokines.

**Figure 3:**
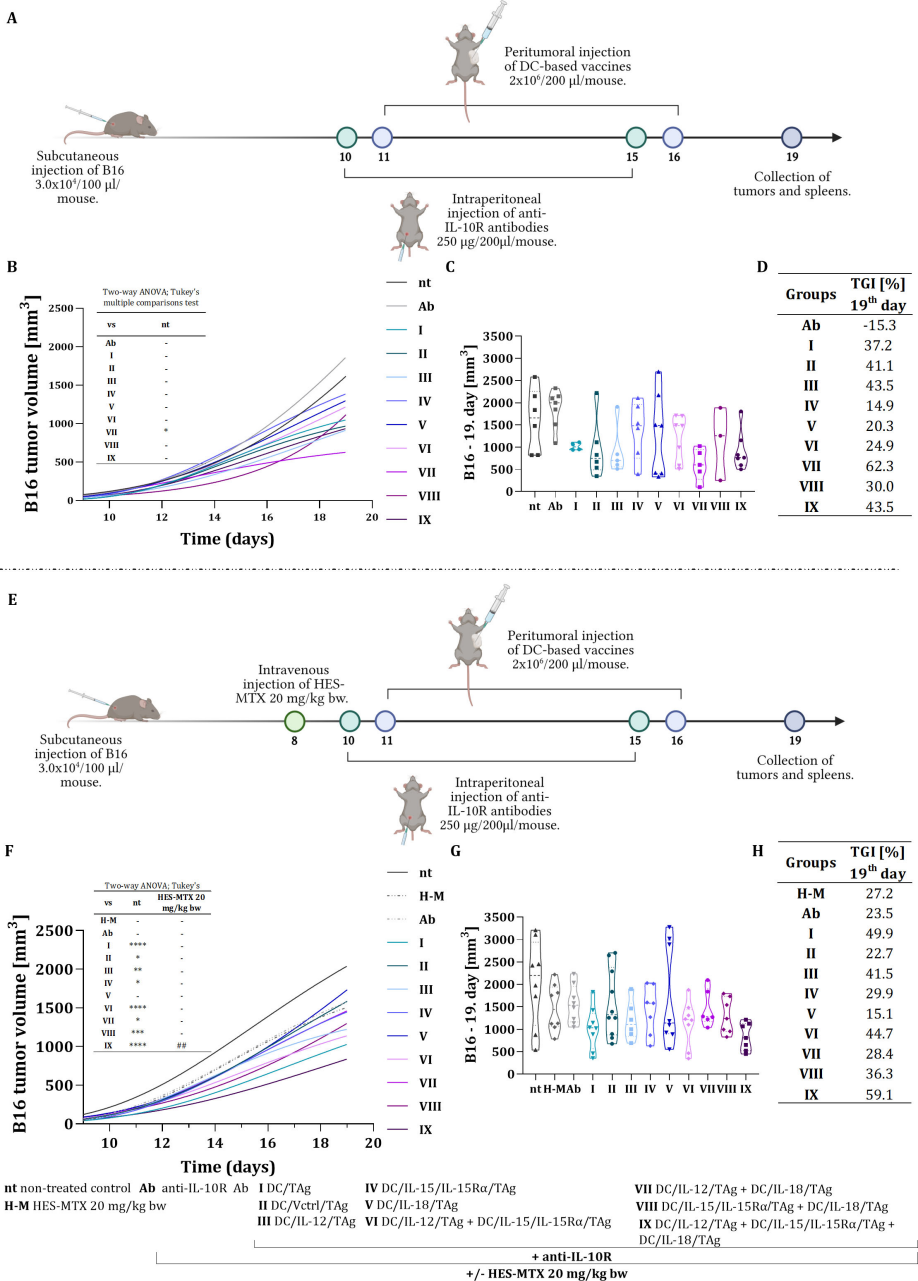
Growth of B16 F0 tumors in mice after immunotherapy and chemoimmunotherapy composed of a chemotherapeutic agent, anti-IL-10R antibody, and genetically modified DC-based vaccines stimulated with B16 F0 tumor antigens. Scheme of immunotherapy **(A)** and chemoimmunotherapy **(E)** treatment created with BioRender.com. Growth kinetics of B16 F0 tumor in mice treated with immunotherapy **(B)** or chemoimmunotherapy **(F)**. Violin plot presenting individual tumor volume and designated median tumor volume for each group, calculated on the 19^th^ day of the immunotherapy **(C)** or chemoimmunotherapy **(G)** experiment. Results are presented as median for 3–10 mice per group. Table presenting B16 F0 tumor growth inhibition (TGI) calculated on the 19^th^ day of the experiment in relation to the non-treated group (nt) **(D, H)**. Differences between groups were estimated using the two−way ANOVA followed by Tukey’s multiple comparisons post−hoc test **(B, F)** or non-parametric Kruskal−Wallis test followed by Dunn’s multiple comparisons test **(C, G)**. The asterisks (*) presented in the graphs indicate statistically significant differences between the given groups and the non-treated control group (nt); hashtags (#) above a bar indicate a statistically significant difference between the given group and the HES-MTX treated group (H-M) – (*p<0.05; **/^##^p<0.01; ***p<0.001; ****p<0.0001).

An above-mentioned *in vitro* study indicated that multi-component dendritic cell-based vaccines can activate splenocytes to produce significant amounts of IFN-γ and increase specific antitumor cytotoxicity, regardless of parallel IL-10 secretion. Therefore, at the outset of our therapeutic experiments using the B16 F0 melanoma model, we aimed to determine whether multi-component vaccines would be more effective in inhibiting tumor growth and initiating immune responses compared to one-component vaccines. Consequently, we applied cellular vaccines in a combined approach of immunotherapy and chemoimmunotherapy for B16 F0 melanoma (**Figures 3A, E**). The effectiveness of both treatments was compared based on the tumor growth rate (**Figures 3B, F**). Treatment outcomes were evaluated on the 19^th^ day of the experiments, where the median tumor volume was illustrated using violin plots (**Figures 3C, G**). Additionally, the tumor growth inhibition rate (TGI) for each treatment group was calculated relative to the untreated control group (**Figures 3D, H**).

The scheme of immunotherapy was presented in **Figure 3A**. To minimize the adverse effects of IL-10 present in the tumor microenvironment, the immunotherapeutic protocol involved administering cellular vaccines after the application of anti-IL-10R antibodies. Mice with subcutaneously growing B16 F0 tumors were treated intraperitoneally with anti-IL-10R antibodies (250 μg/mouse) on the 10^th^ and 15^th^ days of the experiment. One day after the administration of the antibodies, more precisely on the 11^th^ and 16^th^ days of the experiment, cellular vaccines (2 × 10^6^ cells/mouse) were applied peritumorally. DC/TAg (group I) and DC/Vctrl/TAg (group II) were used as control cells.

Analyzing the effects of various components of immunotherapy, we found that the use of anti-IL-10R antibodies alone was not efficient in inhibiting tumor growth. In contrast, the application of DC-based vaccines led to changes in tumor growth inhibition, which varied depending on the cell vaccine type. Notably, among the transduced DCs, the one-component vaccine DC/IL-12/TAg (group III) effectively reduced tumor nodules by 43.5%. These results were comparable to those obtained with the three-component vaccine (group IX), which also demonstrated a 43.5% tumor growth inhibition compared to the untreated group. However, the two-component vaccine combining DC/IL-12/TAg and DC/IL-18/TAg (group VII) achieved the most significant tumor growth inhibition, with a TGI of 62.3% (**Figure 3D**).

The scheme of chemoimmunotherapy was presented in **Figure 3E**. Our previous report demonstrated that administering the HES-MTX nanoconjugate at a dose of 20 mg/kg body weight resulted in beneficial immunomodulation of the antitumor response in a colon cancer model and B16 F0 melanoma model (24–26). Based on this evidence, the current study extended the therapeutic regimen to include the intravenous administration of the HES-MTX nanoconjugate (20 mg/kg body weight) on the 8^th^ day of the experiment.

The administration of HES-MTX prior to immunotherapy enhanced the therapeutic effect across most groups, resulting in higher tumor growth inhibition values compared to immunotherapy alone. The overall effectiveness was more influenced by the type of dendritic cell transduction than by high cytokine secretion. Therefore, the combined therapy, which included a three-component DCs vaccine (DC/IL-12/TAg + DC/IL-15/IL-15Rα/TAg + DC/IL-18/TAg – group IX), achieved the most significant reduction in tumor growth, with an inhibition rate of 59.1% (**Figure 3H**).

Our observation suggests that the effect of immunotherapy did not depend on the high capacity of DCs to produce one particular cytokine, but it was conditioned by the mutual beneficial effect of different cytokines, even if they were secreted by DCs at moderate levels. The use of HES-MTX nanoconjugate resulted in statistically significant differences in tumor growth rate between the untreated control group and the groups administered dendritic cells.

### 3.3 The influence of applied therapies with transduced DCs on immune cell infiltration into B16 F0 tumors

**Figure 4:**
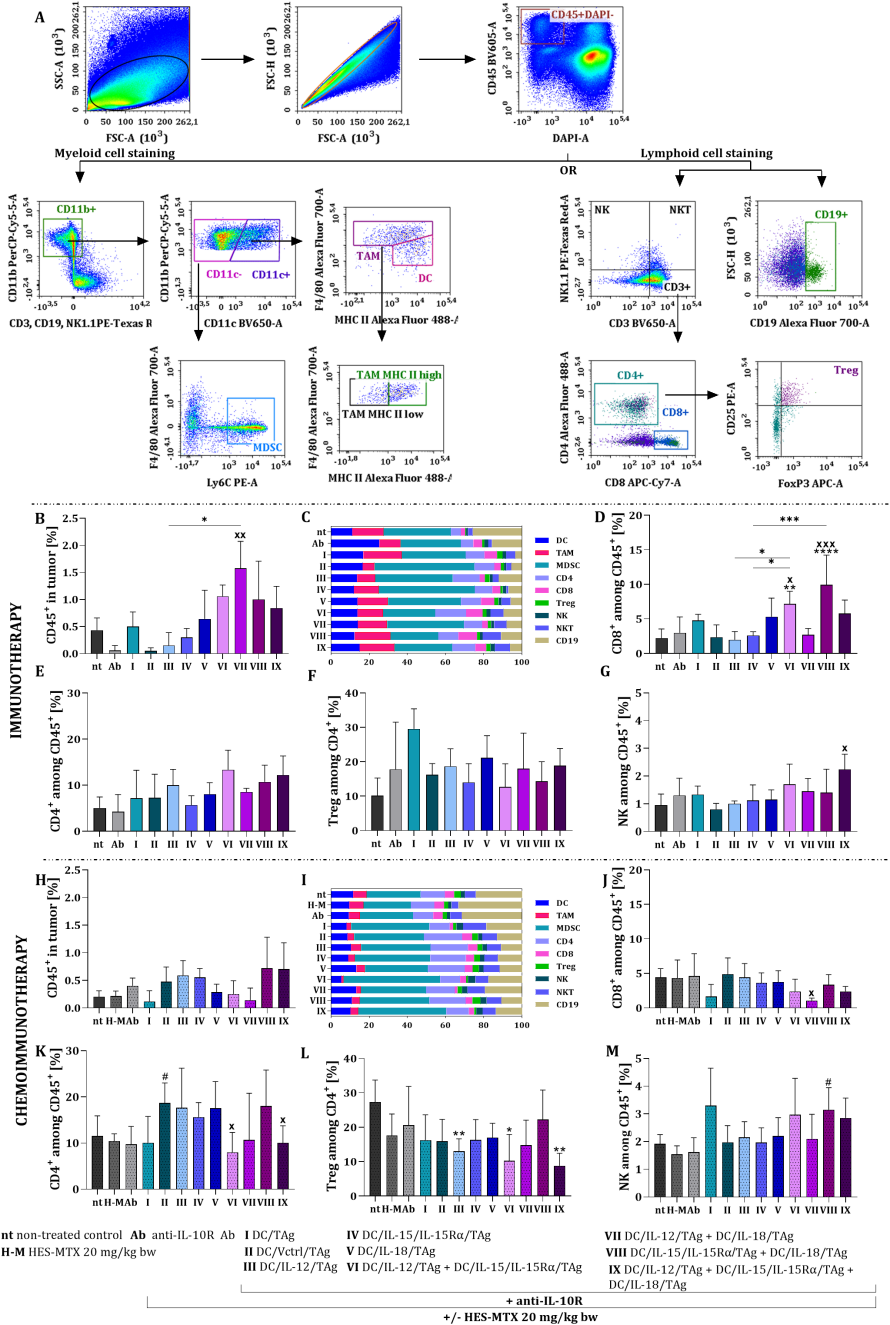
Evaluation of leukocyte subpopulations infiltrating B16 F0 tumor tissue after administration of immunotherapy or chemoimmunotherapy. Scheme of the multiparameter flow cytometry analysis of myeloid and lymphoid cells infiltrating B16 F0 tumor nodules (prepared for one representative sample from group IX in the chemoimmunotherapy experiment) **(A)**. Percentage of live CD45^+^ cells in tumor **(B, H)**. Percentage of each leukocyte’s population among CD45^+^ cells **(C, I)**. Percentage of CD8^+^**(D, J)**, CD4^+^**(E, K)**, Treg **(F, L)** and NK cells **(G, M)** infiltrating tumor tissue. Results are presented as mean+SD calculated for 3–6 mice per group. Differences between groups were estimated using the non-parametric Kruskal-Wallis test followed by Dunn’s multiple comparisons *post-hoc* test **(B, G, J, M)**, the parametric one-way ANOVA followed by Tukey’s multiple comparisons *post-hoc* test **(D)** or the parametric Brown-Forsythe and Welch ANOVA test followed by Dunnett’s T3 multiple comparisons *post-hoc* test **(K, L)**. The asterisks (*) presented in the graphs indicate statistically significant differences between the given groups and the non-treated control group (nt); hashtags (#) above a bar indicate a statistically significant difference between the given group and the HES-MTX treated group **(H-M)**; crosses (X) indicate a statistically significant difference between the given group and the DC/Vctrl/TAg treated group; asterisks (*) under the line indicate statistically significant differences between the given groups – (*/^#^/^x^p<0.05; **/^xx^p<0.01; ***/^xxx^p<0.001; ****p<0.0001).

Tumor nodules were harvested on the 19^th^ day of therapy to identify leukocyte influx. In view of this, we used the extended protocol of a multicolor flow-cytometric analysis to determine the influence of the applied therapies on lymphoid and myeloid cell infiltration into tumors and creation of immune response inside the B16 F0 melanoma microenvironment (**Figure 4A**). Briefly, among alive leukocytes (CD45^+^DAPI^-^), we examined changes in the percentage of particular leukocyte subpopulation such as lymphocytes T CD8 (CD3^+^CD8^+^), T CD4 (CD3^+^CD4^+^), Treg (CD3^+^CD4^+^CD25^+^FoxP3^+^), NK (CD3^-^NK1.1^+^), NKT (CD3^+^NK1.1^+^) and B lymphocytes (CD19^+^) and also myeloid cells (CD11b^+^) including DCs (CD11c^+^F4/80^int^MHC II^+^), TAMs (CD11c^+^F4/80^+^) and MDSCs (CD11c^-^Ly6C^+^). Despite extensive cytometric analysis of cells infiltrating the tumor tissue, we did not notice significant differences in all analyzed immune cell populations. Therefore, we have limited discussion of our results to those immune cell subpopulations with the most meaningful alterations.

We started investigating the impact of immunotherapy by evaluating the changes in the percentage of infiltrating CD45^+^ cells due to various types of vaccines. The lowest percentages of leukocyte influx were observed in the anti-IL-10R Ab and DC/Vctrl/TAg groups. In contrast, the highest leukocyte influx was recorded with the combination of DC/IL-12/TAg and DC/IL-18/TAg (group VII) (**Figure 4B**). The summary plots displaying normalized data helped us visualize the differences among individual leukocyte subpopulations (**Figure 4C**). Analysis of these specific subpopulations confirmed that lymphocytes represented a small portion of the infiltrating leukocytes. A statistically significant increase in the size of this subpopulation was only observed when the DC/IL-12/TAg + DC/IL-15/IL-15Rα/TAg vaccine (group VI) or the DC/IL-15/IL-15Rα/TAg + DC/IL-18/TAg vaccine (group VIII) (**Figure 4D**) were used. The administration of DC-based vaccines led to an increase in the percentage of CD4^+^ cells compared to the non-treated (nt) or antibodies-treated (Ab) groups, however, these changes were not statistically significant (**Figure 4E**). Similarly, in the case of CD4^+^ cells, no significant changes were noted in the regulatory T cell subpopulations relative to either the nt group or the treated DC/TAg vaccine group (**Figure 4F**). Notable changes in the percentage of NK cells among the DC-treated groups were only observed after administering the three-component vaccine (group IX) (**Figure 4G**).

The use of chemoimmunotherapy reduced the percentage of CD45^+^ cells infiltrating tumor tissue, in relation to immunotherapeutic treatment, especially in groups V-VII, making the differences among them unsignificant (**Figure 4H**). Based on the summary plots with normalized data, we observed that the use of HES-MTX affected the increase in the percentage of CD19^+^ cells (**Figure 4I**). In comparison to control group DC/Vctrl/TAg the significant decrease in the percentage of CD8^+^ cells was observed when two-component vaccine DC/IL-12/TAg + DC/IL-18/TAg (group VII) was administered (**Figure 4J**). After the administration of HES-MTX nanoconjugate prior to immunotherapy, there was a strong increase in the percentage of CD4^+^ cells in tumor tissue and this was particularly evident in groups II - V and VIII (**Figure 4K**).

However, it should be highlighted that despite the HES-MTX-induced increased in the size of CD4^+^ T cells population, the percentage of Tregs among CD4^+^ cells was significantly diminished, especially in group receiving DC/IL-12/TAg (group III) and DC/IL-12/TAg + DC/IL-15/IL-15Rα/TAg + DC/IL-18/TAg (group IX) (**Figure 4L**). An increase in the percentage of NK cells was observed in groups I, VI and IX but was significant only after treatment with the DC/IL-15/IL-15Rα/TAg + DC/IL-18/TAg (group VIII) (**Figure 4M**).

**Figure 5:**
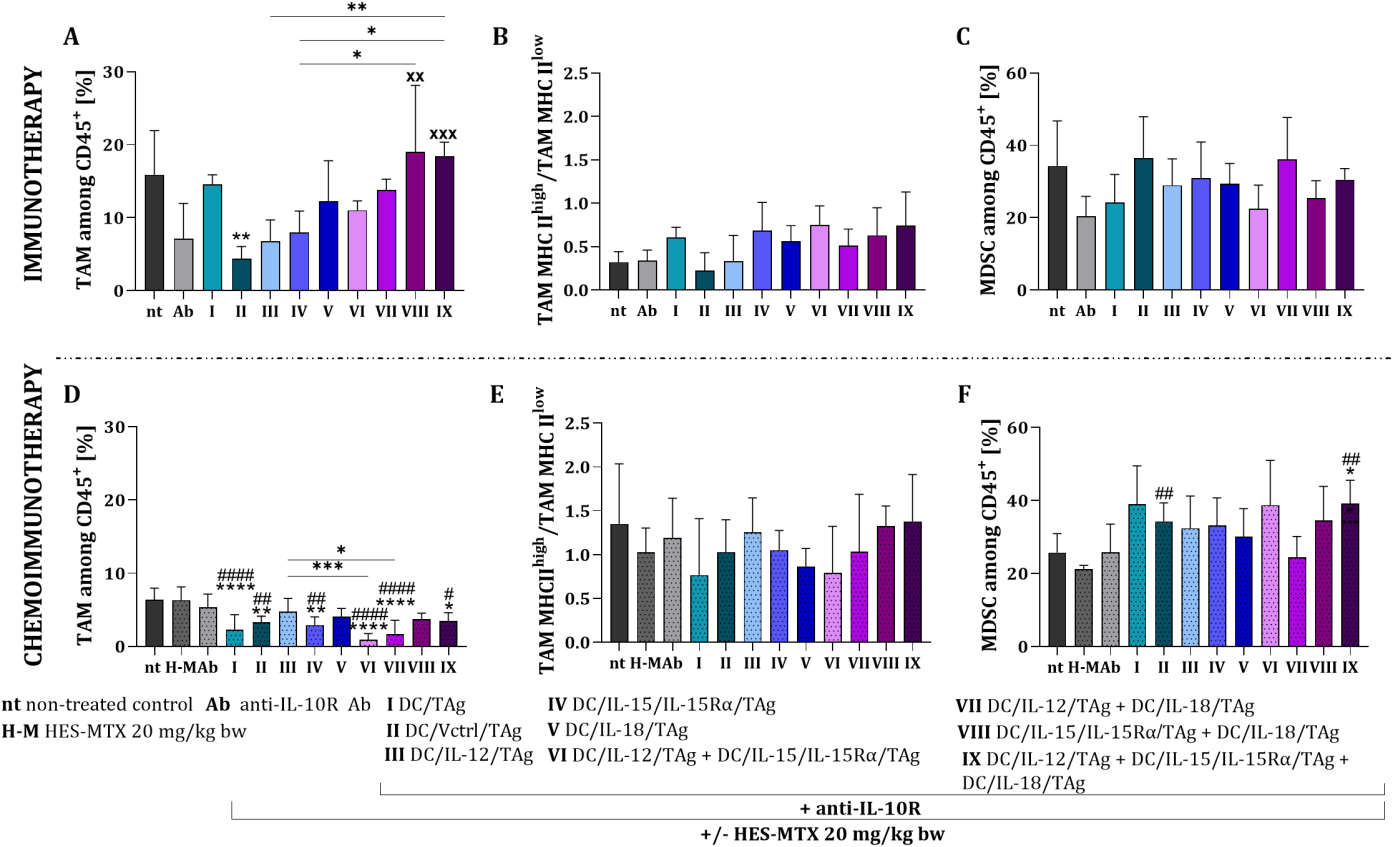
Estimation of TAM and MDSC subpopulation in B16 F0 tumor tissue after applied therapy. Percentage of TAM **(A, D)**, TAMs MHC II^high^/TAMs MHC II^low^**(B, E)** and MDSC **(C, F)** among CD45^+^ cells in tumors. Results are presented as mean+SD calculated for 3–6 mice per group. Differences between groups were estimated using the parametric one-way ANOVA followed by Tukey’s multiple comparisons *post-hoc* test **(A, D)** or the parametric Brown-Forsythe and Welch ANOVA test followed by Dunnett’s T3 multiple comparisons *post-hoc* test **(F)**. The asterisks (*) presented in the graphs indicate statistically significant differences between the given groups and the non-treated control group (nt); hashtags (#) above a bar indicate a statistically significant difference between the given group and the HES-MTX treated group (H-M); crosses (X) indicate a statistically significant difference between the given group and the DC/Vctrl/TAg treated group; asterisks (*) under the line indicate statistically significant differences between the given groups – (*/^#^p<0.05; **/^##^/^xx^p<0.01; ***/^xxx^p<0.001; ****/^####^p<0.0001).

An unexpected effect of immunotherapy with vaccines containing dendritic cell mixtures was observed regarding changes in the size of tumor-associated macrophage (TAM) subpopulations. Specifically, vaccines composed of two or three components increased the percentage of TAMs, whereas control cells (DC/Vctrl/TAg) resulted in a significant decrease in the percentage of TAMs within tumor tissue (**Figure 5A**). When examining the activation status of TAMs in tumors, as indicated by the level of MHC II expression on their surface, we found that TAMs expressing low levels of MHC II predominated. This suggests that the immune response induced by this type of immunotherapy was polarized more towards a Th2 than a Th1 type (**Figure 5B**). Additionally, a high percentage of MDSCs was observed, which exhibited only minor variations regardless of the cytokine present in the environment (**Figure 5C**).

The use of the HES-MTX nanoconjugate in combination with DC-based vaccines significantly reduced the percentage of tumor-associated macrophages compared to the untreated and chemotherapeutically treated groups (**Figure 5D**). The highest reduction of TAM subpopulation was observed after chemoimmunotherapy with DC/IL-12/TAg + DC/IL-15/IL-15Rα/TAg (group VI) or DC/IL-12/TAg + DC/IL-18/TAg (group VII). There was no significant difference between ratio of TAMs with high MHC II expression to cells with low expression (**Figure 5E**). Furthermore, the application of the nanoconjugate prior to most of the DC-based vaccines (except the group treated with DC/IL-12/TAg + DC/IL-18/TAg – group VII) led to an increase in the percentage of myeloid-derived suppressor cells (**Figure 5F**).

Overall, using the nanoconjugate prior to immunotherapy reduced the presence of immunosuppressive cells infiltrating the tumor tissue, specifically Treg and TAMs. The most significant effects were observed following the administration of the two-component vaccine (DC/IL-12/TAg + DC/IL-15/IL-15Rα/TAg – group VI) and the three-component vaccine.

### 3.4 Effect of multicomponent therapy on the activation of the systemic antitumor response

**Figure 6:**
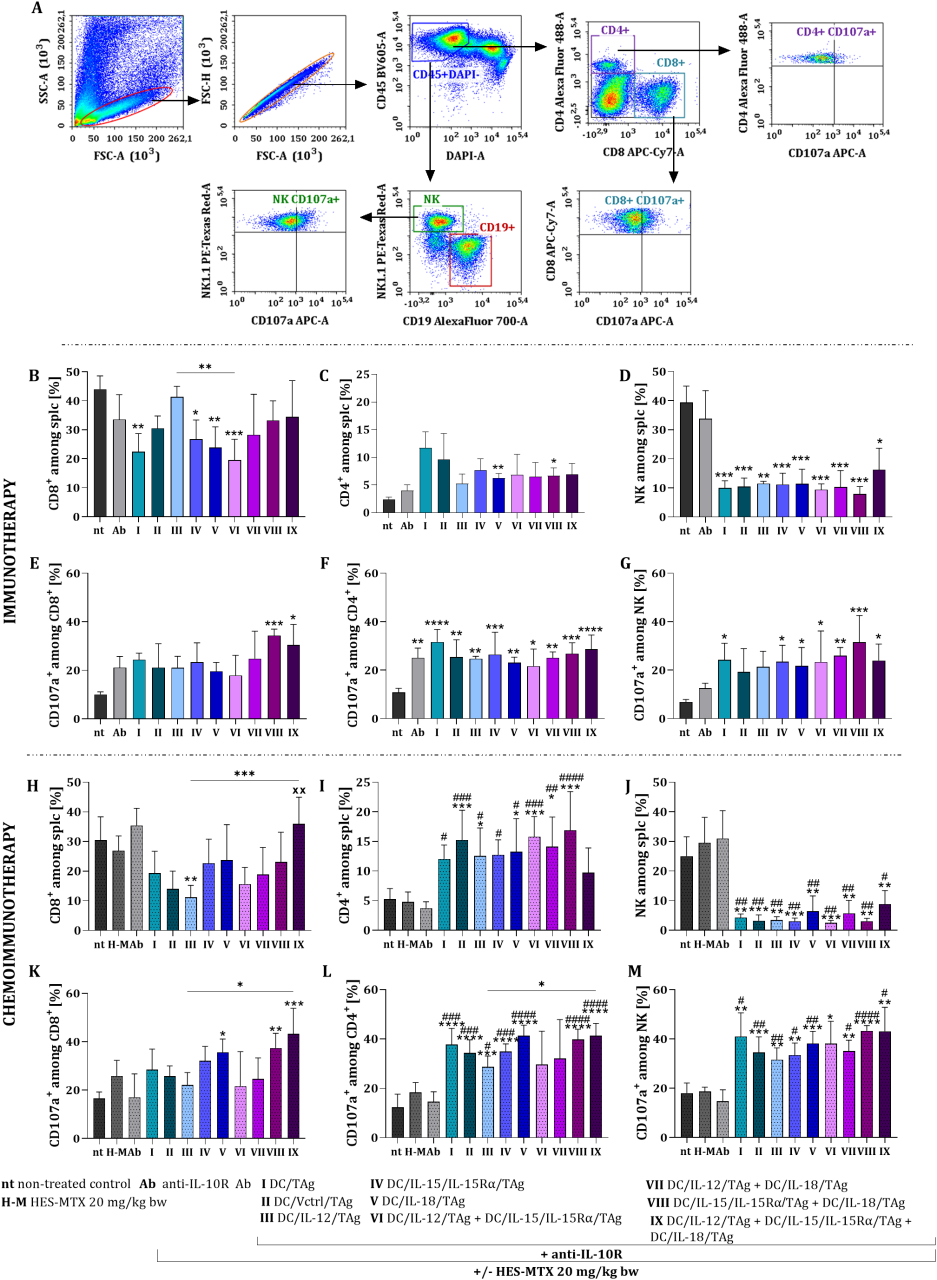
Impact of applied immunotherapy and chemoimmunotherapy on the induction of systemic antitumor in B16 F0 melanoma model. Scheme of the flow cytometry analysis of restimulated splenocytes **(A)**. Percentage of CD8^+^**(B, H)**, CD4^+^**(C, I)**, and NK cells **(D, J)** restimulated splenocytes. Percentage of CD107a^+^ cells among CD8^+^**(E, K)**, CD4^+^**(F, L)** and NK cells **(G, M)**. Results are presented as mean+SD calculated for 3–6 mice per group. Differences between groups were estimated using the non-parametric Kruskal-Wallis test followed by Dunn’s multiple comparisons *post-hoc* test **(E, H, K)**, the parametric one-way ANOVA followed by Tukey’s multiple comparisons *post-hoc* test **(B, F, G, I)** or the parametric Brown-Forsythe and Welch ANOVA test followed by Dunnett’s T3 multiple comparisons *post-hoc* test **(C, D, J, L, M)**. The asterisks (*) presented in the graphs indicate statistically significant differences between the given groups and the non-treated control group (nt); hashtags (#) above a bar indicate a statistically significant difference between the given group and the HES-MTX treated group **(H-M)**; crosses (X) indicate a statistically significant difference between the given group and the DC/Vctrl/TAg treated group; asterisks (*) under the line indicate statistically significant differences between the given groups – (*/^#^p<0.05; **/^##^/^xx^p<0.01; ***/^###^p<0.001; ****/^####^p<0.0001).

In both therapies, we assessed the impact of DC-based vaccines on the activation of systemic antitumor responses. Specifically, spleens from B16 F0-bearing mice that had been treated were harvested on the 19^th^ day and restimulated ex vivo with B16 F0 cells in a 5-day mixed culture. Live splenocytes were then analyzed using multiparameter flow cytometry to determine the changes in the percentages of CD8^+^, CD4^+^, and NK cells, including the proportion of effector cells (identified by the expression of CD107a molecules) (**Figure 6A**).

Among restimulated splenocytes obtained from immunotherapy-treated mice, all types of DC-based vaccines resulted in a decrease in the percentage of CD8^+^ cells compared to the non-treated control group (**Figure 6B**). Notably, in the groups treated with DC/TAg, DC/IL-15/IL-15Rα/TAg, DC/IL-18/TAg, and DC/IL-12/TAg + DC/IL-15/IL-15Rα/TAg (groups I, IV, V, and VI, respectively), the percentage of CD8^+^ cells was significantly lower than that of the non-treated group.

However, it was observed that the use of DC-based vaccines, particularly DC/IL-18/TAg (group V) and DC/IL-15/IL-15Rα/TAg + DC/IL-18/TAg (group VIII), led to an increase in the percentage of CD4^+^ cells among all restimulated splenocytes compared to the untreated group (**Figure 6C**). Additionally, the percentage of NK cells among restimulated splenocytes reached almost 40% in the non-treated and antibody-treated groups. In contrast, all groups that underwent DC-based immunotherapy exhibited a significant reduction in the size of this subpopulation (**Figure 6D**).

It is important to note that each type of DC-based vaccine treatment significantly increased the percentage of CD107a^+^ cells compared to the non-treated control group. Among the treatments evaluated, a vaccine containing DC/IL-15/IL-15Rα/TAg + DC/IL-18/TAg (group VIII) was the most promising in activating CD8^+^ (**Figure 6E**) and NK cells (**Figure 6F**), both of which are capable of releasing cytolytic granules. Additionally, the restimulated CD4^+^ cells from B16 F0-bearing mice showed an increased percentage of CD107a^+^ cells following every type of treatment, indicating an enhancement in the cytotoxic capacity of these cells (**Figure 6G**).

Similar to the immunotherapy regimen, chemoimmunotherapy resulted in a decrease in the percentage of restimulated CD8^+^ cells in almost all DC-treated groups, especially after treatment with the DC/IL-12/TAg vaccine (group III) (**Figure 6H**). However, the three-component vaccine enhanced the growth of the CD8^+^ subpopulation compared to the DC/Vctrl/TAg control (group II). An opposing trend was observed regarding the CD4^+^ cell subpopulation after treatment with all vaccine types. This increase was particularly significant after the administration of DC/Vctrl/TAg (group II), DC/IL-12/TAg + DC/IL-15/IL-15Rα/TAg (group VI), and DC/IL-15/IL-15Rα/TAg + DC/IL-18/TAg (group VIII) (**Figure 6I**).

In all groups receiving cell vaccines, a decrease in the number of NK cells among splenocytes was observed compared to all control groups (groups: nt, H-M, Ab) (**Figure 6J**). The increase in the percentage of CD107a^+^ cells within the CD8^+^ cell population was observed following the administration of DC/IL-18/TAg (group V), the two-component vaccine DC/IL-15/IL-15Rα/TAg + DC/IL-18/TAg (group VIII) and the three-component vaccine (group IX) (**Figure 6K**). This suggests that primarily IL-18 may be responsible for the increased size of CD8^+^CD107a^+^ spleen cell subpopulation. In contrast to that subpopulation, the percentage of CD107a^+^CD4^+^ and CD107a^+^NK cells among splenocytes increased in groups receiving cell vaccines, regardless of their type (**Figures 6L, M**).

**Figure 7:**
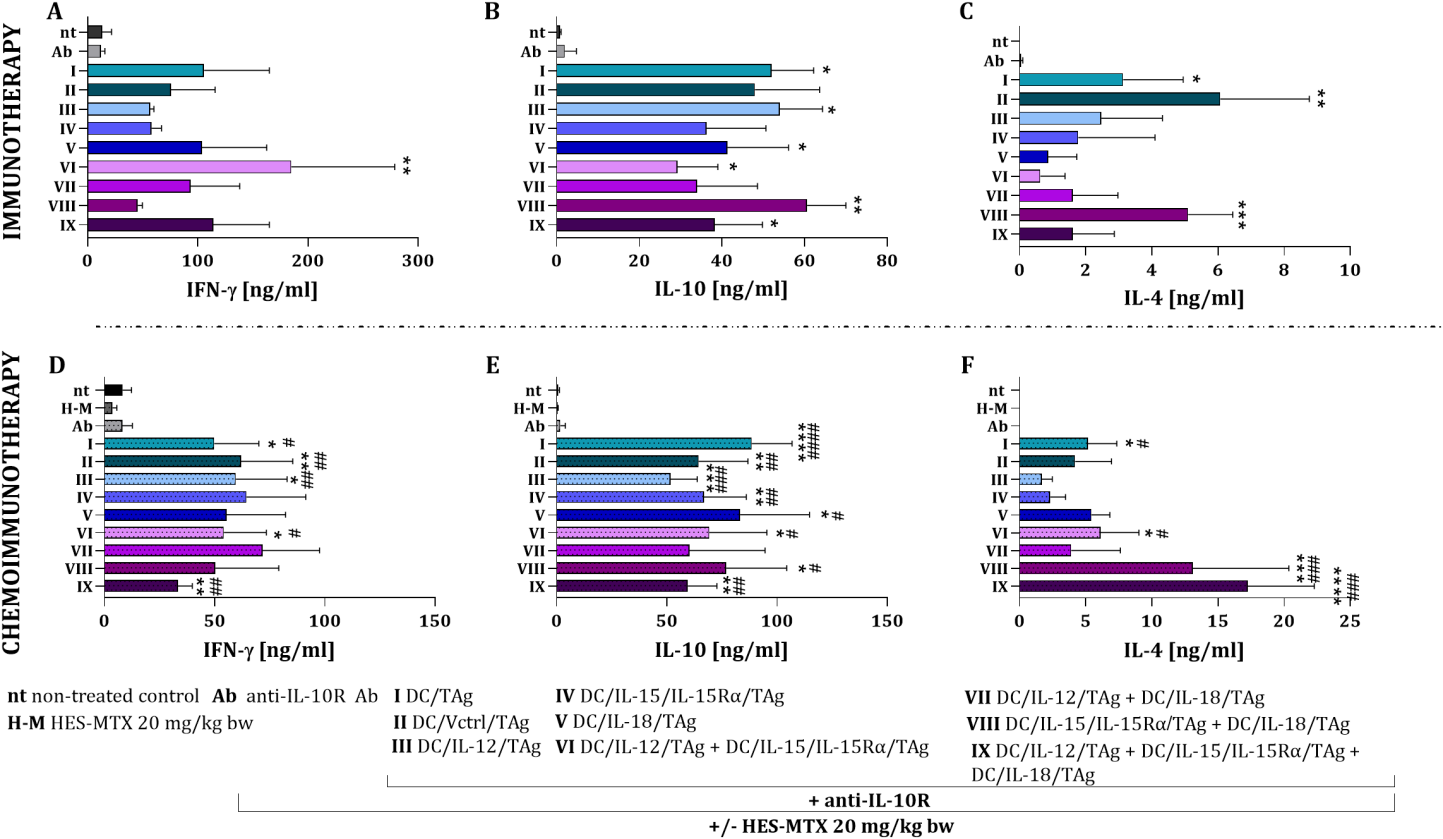
Activity of restimulated splenocytes obtained from B16 F0 melanoma-bearing mice after immunotherapy and chemoimmunotherapy. Concentration of IFN-γ **(A, D)**, IL-10 **(B, E)** and IL-4 **(C, F)** in supernatants after restimulation of spleen cells with B16 F0 cells, measured using ELISA assay. Results are presented as mean+SD calculated for 3–6 mice per group. Differences between groups were estimated using the non-parametric Kruskal-Wallis test followed by Dunn’s multiple comparisons *post-hoc* test **(A, C, F)** or the parametric Brown-Forsythe and Welch ANOVA test followed by Dunnett’s T3 multiple comparisons *post-hoc* test **(B, D, E)**. The asterisks (*) presented in the graphs indicate statistically significant differences between the given groups and the non-treated control group (nt); hashtags (#) above a bar indicate a statistically significant difference between the given group and the HES-MTX treated group (H-M); crosses (X) indicate a statistically significant difference between the given group and the DC/Vctrl/TAg treated group; asterisks (*) under the line indicate statistically significant differences between the given groups – (*/^#^p<0.05; **/^##^p<0.01; ***/^###^p<0.001; ****/^####^p<0.0001).

To confirm the effectiveness of the antitumor response activated by transduced DCs, we examined the ability of restimulated splenocytes to produce cytokines. In the context of immunotherapy, all restimulated splenocytes obtained from the treated groups (groups I-IX) demonstrated high production levels of IFN-γ, IL-10, and IL-4. Notably, splenocytes from mice treated with the combination of DC/IL-12/TAg + DC/IL-15/IL-15Rα/TAg (group VI) produced the highest amounts of IFN-γ (approx. 185 ng/ml) (**Figure 7A**). While the levels of IL-10 were similar across all groups, the vaccine consisting of two components, DC/IL-15/IL-15Rα/TAg + DC/IL-18/TAg (group VIII), induced the highest production of this cytokine (approx. 61 ng/ml) (**Figure 7B**). The highest levels of IL-4 were observed in the groups receiving either DC/TAg (group I, approx. 3.1 ng/ml), DC/Vctrl/TAg (group II, approx. 6.1 ng/ml) or the combination of DC/IL-15/IL-15Rα/TAg + DC/IL-18/TAg (group VIII) (**Figure 7C**, approx. 5.1 ng/ml). Overall, splenocytes from mice treated with dendritic cells that overproduce IL-12 were more efficient in producing IFN-γ, while the highest production of anti-inflammatory cytokines was observed following the administration of the vaccine DC/IL-15/IL-15Rα/TAg + DC/IL-18/TAg (group VIII).

The use of HES-MTX nanoconjugate before immunotherapy resulted in increased production of IFN-γ (**Figure 7D**) and IL-10 (**Figure 7E**) by restimulated splenocytes from all groups receiving cellular vaccines. However, there were no statistically significant differences between these groups. The HES-MTX nanoconjugate also influenced changes in the production of IL-4. The highest ability to produce this cytokine was characterized by splenocytes obtained from mice treated with combination therapy including DC/IL-15/IL-15Rα/TAg + DC/IL-18/TAg (group VIII, approx. 13.1 ng/ml) and three-component vaccine of DC/IL-12/TAg + DC/IL-15/IL-15Rα/TAg + DC/IL-18/TAg (group IX, approx. 17.2 ng/ml) (**Figure 7F**).

## 4 Summary

The use of the vaccine preparation scheme developed in previous studies (26) allowed to obtain modified DCs capable of secreting IL-12, IL-15/IL-15Rα, IL-18 and stimulating a specific immune response against B16 F0 melanoma cells *in vitro*.

The use of anti-IL-10R antibodies and DC-derived vaccines in immunotherapy of B16 F0 melanoma led to inhibition of tumor growth. However, its rate of change depended on the type of vaccine used. Significant inhibition of tumor growth was observed after administering vaccines composed of DC/IL-12/TAg combined with DC/IL-18/TAg (group VII), resulting in a tumor growth inhibition of 62.5%. In contrast, administration of anti-IL-10R antibodies alone accelerated the growth rate of B16 F0 tumors.

The sole use of HES-MTX at a dose of 20 mg/kg caused a slight inhibition of the B16 F0 tumors growth rate. Its application in combination with the anti-IL-10R antibody affected a slight increase in the percentage of CD45^+^ cells infiltrating the tumor tissue. The therapy supplementation with vaccines additionally strengthened this effect and the highest percentage of infiltrating cells was observed in groups of mice that received the two-component vaccine DC/IL-15/IL-15Rα/TAg + DC/IL-18/TAg (group VIII) and the three-component vaccine (group IX) (**Table 2**). Analyzing the division of this leukocyte subpopulation, we revealed that the chemoimmunotherapy reduced the percentage of CD8^+^ and CD4^+^ cells among CD45^+^ cells compared to immunotherapy, but also decreased the percentage of suppressor regulatory T cells among CD4^+^ cells and tumor-associated macrophages among the total CD45^+^ cell population.

**Table 2:** Leukocytes infiltration into B16 F0 tumor nodule after immunotherapy (I) and chemoimmunotherapy (CI) shown as the ratio of the average values ​​of a given subpopulation to the average value of the untreated group.

Type of therapy	CD45^+^ among tumor nodule		CD8^++^ among CD45^+^		CD4^+^ among CD45^+^		Treg among CD4^+^		NK among CD45^+^		TAM among CD45^+^		TAM MHC II^high^/TAM MHC II^low^		MDSC among CD45^+^	
	I	CI	I	CI	I	CI	I	CI	I	CI	I	CI	I	CI	I	CI
H-M		1.1		1.0		0.9		0.6		0.8		1.0		0.8		0.8
Ab	0.1	2.0	1.4	1.0	0.8	0.8	1.8	0.8	1.4	0.8	0.4	0.8	1.1	0.9	0.6	1.0
I	1.2	0.6	2.2	0.4	1.4	0.9	2.9	0.6	1.4	1.7	0.9	0.4	1.9	0.6	0.7	1.5
II	0.1	2.4	1.1	1.1	1.4	1.6	1.6	0.6	0.8	1.0	0.3	0.5	0.7	0.8	1.1	1.3
III	0.3	2.9	0.9	1.0	2.0	1.5	1.8	0.5	1.1	1.1	0.4	0.7	1.0	0.9	0.8	1.3
IV	0.7	2.8	1.2	0.8	1.1	1.4	1.4	0.6	1.2	1.0	0.5	0.5	2.1	0.8	0.9	1.3
V	1.5	1.4	2.4	0.8	1.6	1.5	2.1	0.6	1.2	1.1	0.8	0.6	1.8	0.6	0.9	1.2
VI	2.4	1.3	3.3	0.5	2.7	0.7	1.2	0.4	1.8	1.5	0.7	0.1	2.3	0.6	0.7	1.5
VII	3.6	0.7	1.2	0.2	1.7	0.9	1.8	0.5	1.5	1.1	0.9	0.3	1.6	0.8	1.0	0.9
VIII	2.3	3.6	4.5	0.8	2.1	1.6	1.4	0.8	1.5	1.6	1.2	0.6	2.0	1.0	0.7	1.3
IX	1.9	3.5	2.6	0.5	2.4	0.9	1.9	0.3	2.4	1.5	1.2	0.5	2.3	1.0	0.9	1.5

H-M, HES-MTX; Ab, anti-IL-10R antibody; I, DC/TAg; II, DC/Vctrl/TAg; III, DC/IL-12/TAg; IV, DC/IL-15/IL-15Rα/TAg; V, DC/IL-18/TAg; VI, DC/IL-12/TAg + DC/IL-15/IL-15Rα/TAg; VII, DC/IL-12/TAg + DC/IL-18/TAg; VIII, DC/IL-15/IL-15Rα/TAg + DC/IL-18/TAg; IX, DC/IL-12/TAg + DC/IL-15/IL-15Rα/TAg + DC/IL-18/TAg.Colors represent relative values within the selected range: green indicates higher values, red indicates lower values, with color intensity reflecting magnitude.

The analysis of restimulated splenocyte activity indicated that the combination of HES-MTX nanoconjugate, anti-IL-10R antibodies, and cell vaccines led to an increase in the percentage of CD107a^+^ cells within the CD4^+^ cell population. However, there was a slight decrease in the percentage of CD107a^+^ cells among the CD8^+^ and NK cell populations compared to sole immunotherapy (**Table 3**).

**Table 3:** Analysis of restimulated splenocytes obtained after immunotherapy (I) and chemoimmunotherapy (CI) presented as the ratio of the average values of a given subpopulation to the average value of the untreated group.

Type of therapy	CD4^+^ among splc		CD8^+^ among splc		NK among splc [%]		CD107a^+^ among CD4^+^		CD107a^+^ among CD8^+^		CD107a^+^ among NK	
	I	CI	I	CI	I	CI	I	CI	I	CI	I	CI
H-M		0.9		0.9		1.2		1.5		1.6		1.0
Ab	1.7	0.7	0.8	1.2	0.9	1.2	2.3	1.2	2.1	1.0	1.8	0.8
I	4.9	2.3	0.5	0.6	0.3	0.2	2.9	3.1	2.4	1.7	3.5	2.3
II	4.0	2.9	0.7	0.5	0.3	0.1	2.3	2.8	2.1	1.6	2.8	1.9
III	2.2	2.4	0.9	0.4	0.3	0.1	2.3	2.3	2.1	1.3	3.1	1.8
IV	3.2	2.4	0.6	0.7	0.3	0.1	2.4	2.8	2.3	1.9	3.4	1.9
V	2.6	2.5	0.5	0.8	0.3	0.3	2.1	3.4	2.0	2.2	3.1	2.1
VI	2.9	3.0	0.4	0.5	0.2	0.1	2.0	2.4	1.8	1.3	3.4	2.1
VII	2.7	2.7	0.6	0.6	0.3	0.2	2.3	2.6	2.5	1.5	3.8	1.9
VIII	2.8	3.2	0.8	0.8	0.2	0.1	2.5	3.2	3.4	2.3	4.6	2.4
IX	2.9	1.8	0.8	1.2	0.4	0.4	2.6	3.4	3.0	2.6	3.5	2.4

H-M, HES-MTX; Ab, anti-IL-10R antibody; I, DC/TAg; II, DC/Vctrl/TAg; III, DC/IL-12/TAg; IV, DC/IL-15/IL-15Rα/TAg; V, DC/IL-18/TAg; VI, DC/IL-12/TAg + DC/IL-15/IL-15Rα/TAg; VII, DC/IL-12/TAg + DC/IL-18/TAg; VIII, DC/IL-15/IL-15Rα/TAg + DC/IL-18/TAg; IX, DC/IL-12/TAg + DC/IL-15/IL-15Rα/TAg + DC/IL-18/TAg.Colors represent relative values within the selected range: green indicates higher values, red indicates lower values, with color intensity reflecting magnitude.

We conclude that the treatment of B16 F0 tumor with HES-MTX nanoconjugate, anti-IL-10R antibodies, and a three-component cell vaccine, caused strong tumor growth inhibition by eliciting both local and systemic antitumor responses. This was related to a decrease in the percentage of regulatory T cells among CD4^+^ cells and tumor-associated macrophages among CD45^+^ cells.

**Figure 8:**
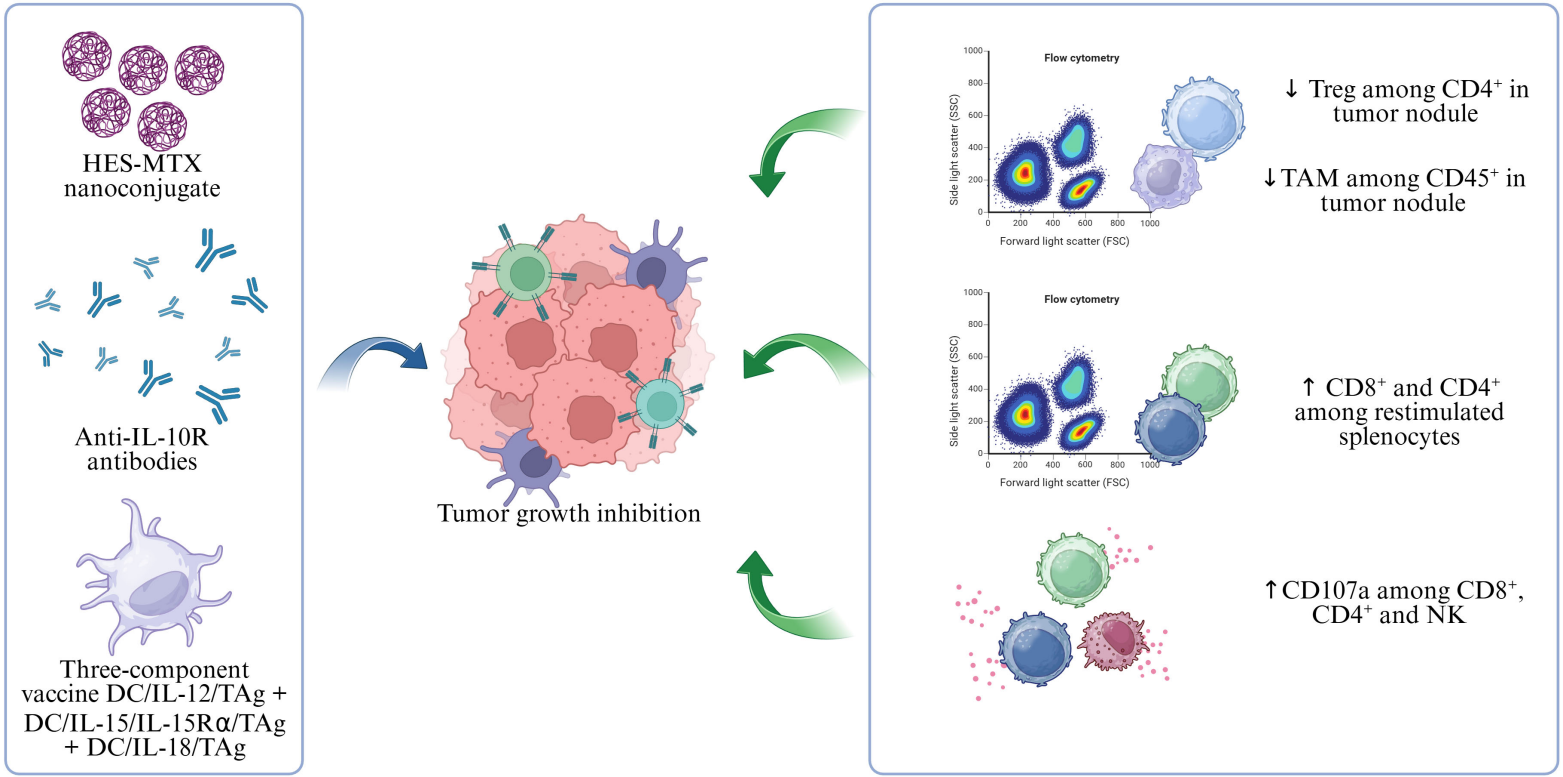
The effect of chemoimmunotherapy with DC/IL-12/TAg + DC/IL-15/IL-15Rα/TAg + DC/IL-18/TAg cell vaccine on the inhibition of B16 F0 tumor growth.

Additionally, there was an observed increase in the percentages of CD4^+^ and CD8^+^ cells among the restimulated splenocytes, along with heightened activity indicated by an increase in the percentage of CD107a-positive cells. The restimulated splenocytes also demonstrated a robust capacity for producing interferon-γ (**Figure 8**).

## 5 Discussion

Our study aimed to determine the effectiveness of the immunotherapy and chemoimmunotherapy regimens in B16 F0 melanoma-bearing mice. These treatment regimens were previously applied to the MC38 mouse model of colon cancer, where promising results were achieved in the chemoimmunotherapy experiment (26). Given that melanoma is a rapidly developing tumor (30), we decided to administer both antibodies and cell vaccines twice, concluding the experiment on the 19^th^ day after the administration of B16 F0 tumor cells.

In the immunotherapy, tumor-bearing mice were injected intraperitoneally with anti-IL-10R antibodies to block the IL-10 receptor on immune cells, thereby minimizing the negative effects of this cytokine. It was agreed with the study of García-Hernández et al. who demonstrated that IL-10 could promote tumor growth in a B16-melanoma model by stimulating tumor cell proliferation, angiogenesis, and immunosuppression (31). Moreover Markham’s team showed that neutralizing IL-10 enhances the anti-tumor efficacy of a MIP3α-gp100 DNA vaccine caused significantly smaller tumors, slower tumor growth, and increased survival rates in mice (32). Furthermore, blocking IL-10 signaling during immunization can facilitate the development of potent vaccines that effectively inhibit tumor growth (7, 33).

Combining anti-IL-10R-mediated signal blockade with DC-based vaccines triggered an immune response, depending on the cytokines secreted by transductants. The administration of these cells overproducing IL-12 resulted in a 43.5% inhibition of tumor growth, whereas using DC/IL-18/TAg was only 20.3%. Meantime, when cells overproducing both IL-12 and IL-18 were mixed in a 1:1 ratio, despite lower amounts of particular factors, the tumor growth inhibition reached 62.3%. The strong inhibition of tumor growth observed after administration of the two-component vaccine might be due to a significant influx of CD45^+^ cells into the tumor. This study and previous research on the colon cancer MC38 (19, 26) indicate that combinations of cytokines yield much better results. Choi et al. investigated the therapeutic potential of an oncolytic adenovirus (Ad) that co-expresses IL-12 and IL-18. Their study demonstrated that the combination of RdB/IL-12/IL-18 resulted in enhanced and prolonged suppression of tumor growth, as well as increased survival rates, compared to either RdB/IL-12 or RdB/IL-18 used alone (34). Similarly, Tatsumi et al. showed that dendritic cells co-producing IL-12 and IL-18 achieved complete tumor rejection by generating an effective Th1 immune response in a BALB/c sarcoma model (35). In our research, we use dendritic cells as carriers of cytokines that gradually release them into the environment, thereby limiting the systemic toxicities. Jones et al. also wanted minimalize the side effect of therapy with IL-12. They used direct tethering of the cytokine to the surface of tumor-specific T cells to control cytokine dose. Scientists have proven the effectiveness and safety of such modification (36). Ghasemi et al. used DC progenitors (DCPs) modified to produce immunostimulatory cytokines, IL-12 and FLT3L. Cytokine-armed DCPs suppressed tumor growth, including melanoma, which was associated with natural killer and T cell infiltration and activation, M1-like macrophage programming and tumor necrosis (37). Gilmour et al. stimulated human dendritic cells with PPR combinations to increase IL-12 production, intending to use them in future anticancer therapies (38). To achieve an effective antitumor response, it is essential to stimulate dendritic cells with tumor antigens. In our studies, we used lysate from B16 F0 cells, but scientists have different approaches. Adoptive therapy combining multi-antigen-loaded dendritic cells and cytokine-induced killer cells (Ag-DC-CIK) represent a promising tool in the fight against solid tumors. Ag-DC-CIK treatment enhanced cytotoxicity, increased apoptosis induction and effectively suppressed tumor cell migration (39). In glioblastoma, the phase III DCVax-L trial showed a survival benefit for patients treated with autologous tumor lysate-loaded dendritic cells. Median overall survival in the DCVax-L group approached two years (40).

In order to evaluate the possibility of combining the above-described dendritic cells-based vaccines together with HES-MTX nanoconjugate chemotherapy, at the beginning, we compare the different doses of MTX and HES-MTX nanoconjugate, i.e., in the B16 mouse melanoma model. In this previous work, we assessed the immunomodulatory effect induced by these chemotherapeutics applied in the dose of 20 mg/kg body weight on the 3^rd^ and 10^th^ day after their administration. We found that on the 3^rd^ day after HES-MTX injection, at the local immune response level, the most significant changes in TME concerned a decrease in the TAM infiltration and an increase in the degree of their activation status at the same time. Moreover we observed a reduction in the size of the Treg cell population in the tumor, with a simultaneous increased total influx of CD4^+^ cells into the tumor nodules. Despite further tumor development, some of these changes were maintained and an increased in influx of DCs into the tumor tissue was also found. Considering the systemic anti-tumor immune response, we proved that shortly after HES-MTX administration the percentages of CD8^+^ and CD4^+^ effector cells (CD44^+^CD62L^neg^) were decreased, but despite this, these re-stimulated spleen cells was characterized with the highest percentage of CD8^+^ T cells (on the 3^rd^ day of therapy) and CD4^+^ T cells (on the 10^th^ day of therapy) (41).

This knowledge gained about the immunomodulatory potential of HES-MTX in B16 melanoma model allowed us to fully explain the observations noticed in the chemoimmunotherapy regimen. Three days after administration of the nanoconjugate (20 mg/kg b.w.), favorable changes occurred in the tumor environment and the systemic immune response. Starting immunotherapy at this time point would enable the development of an immune response activated by cytokine-producing DCs.

Supplementing the therapy with a single administration of the HES-MTX nanoconjugate gives divergent effects that were closely associated with cytokine composition generated by applied DC-based vaccines. The most pronounced reduction effectiveness of DC-based vaccines after administration of HES-MTX was observed in Group VII, which combines DC/IL-12/TAg and DC/IL-18/TAg. This regimen strongly promotes Th1 polarization and IFN-γ–dependent cytotoxic responses (19, 42) and achieved the highest TGI under immunotherapy alone. However, in the chemoimmunotherapy scheme, the final therapeutic outcome was abolished. We conclude that this was closely related with reduction in the population size of effector CD4^+^ and CD8^+^ cells shortly after application of the nanoconjugate (41), and full range of efficacy of this type of DC-vaccines was also limited.

In contrast, treatment regimens containing IL-15 (groups: IV, VI, VIII, and IX) demonstrated preserved or even enhanced antitumor efficacy following HES-MTX administration. IL-15 is known to support CD8^+^ T-cell and NK-cell survival (43) and may therefore stabilize immune responses during or after HES-MTX administration. This stabilizing effect may explain why the triple-cytokine vaccine (group IX, TGI 59.1%) benefited from HES-MTX, whereas the IL-12/IL-18 (group VII, TGI 28.4%) combination alone did not. In comparison, during immunotherapy with the three-component vaccine, tumor growth inhibition was 43.5%. A similar effect was noted in mice with colon cancer MC38. Immunotherapy alone resulted in a 24.0% TGI, whereas the combination of chemo- and immunotherapy led to a TGI of 69.9% (26). Gao-Na Shi et al. suggested that methotrexate could enhance antigen presentation and T cell priming, making it a potential adjuvant in dendritic cell therapies (44).

The use of HES-MTX nanoconjugate increased the influx of CD45^+^ cells in almost all groups receiving cell vaccines. However, this was most pronounced in groups DC/IL-15/IL-15Rα/TAg, + DC/IL-18/TAg and DC/IL-12/TAg + DC/IL-15/IL-15Rα/TAg + DC/IL-18/TAg compared to the untreated control. Previous studies have demonstrated that the use of HES-MTX leads to a significant reduction in the percentages of TAMs and Tregs, with noticeable effects observed as early as the third day of treatment (41). This reduction may have positively impacted therapy outcomes, as elevated levels of both Tregs and TAMs are associated with poorer prognoses (45, 46). Consequently, we chose to begin administering cell vaccines three days after introducing the chemotherapy drug to ensure that the activation of immune cells by dendritic cells was not inhibited by suppressor cells. Another subpopulation prevalent in the B16 melanoma microenvironment is MDSCs. Our previous research indicated that their numbers are even three times higher than those observed in the MC38 tumor model which influences the overall anti-tumor response (25). However, the therapies employed in our study did not affect the size of this subpopulation.

Both immuno- and chemoimmunotherapy led to an increase in the percentage of CD4^+^ cells and a decrease in the influx of CD8^+^ cells and NK cells among restimulated splenocytes, which is typical for our cell vaccines (24, 26). However, an increase in the lymphocyte degranulation marker CD107a was observed on the surface of CD4^+^ cells, particularly following chemoimmunotherapy. Clinic observations and studies in mice suggest that helper CD4^+^ cytotoxic lymphocytes (ThCTL) infiltrating tumor tissue may eliminate some tumor-infiltrating and melanoma tumor cells (47, 48). Different CD4 CTL subsets might likely be induced in response to viral infections and anti-tumor immunity, and their CTL activity is associated, i.a., with CD107a expression. On the one hand, CD4 CTLs can affect cytolytic activity towards melanoma cells, thereby supporting CD8^+^ cells in tumor clearance. However, it can dampen cytotoxic antitumor activity of CD8^+^ cells by secreting the anti-inflammatory cytokine, e.g., IL-10, as it was described in hepatocellular carcinoma (49). We also see both an increase in CD4^+^ cell activation, increased production of IFN-γ but also IL-10 and IL-4. Similar conclusions about the direct cytotoxic potential of CD4^+^ T cells has been shown also in patients with melanomas (50, 51) therefore the observations presented in this work are consistent with other scientific reports.

In summary, the greatest inhibition of tumor growth (62.3%) was observed following immunotherapy with the DC/IL-12/TAg + DC/IL-18/TAg vaccine, we believe this is primarily due to an increased influx of CD45^+^ cells into the tumors. However, when examining individual populations, we do not see significant differences between the groups. A slightly lower tumor growth inhibition (59.1%), was achieved through chemoimmunotherapy with the three-component vaccine DC/IL-12/TAg + DC/IL-15/IL-15Rα/TAg + DC/IL-18/TAg. In this group, we noted an increased influx of CD45^+^ cells accompanied by a decrease in the percentage of Treg and TAMs suppressor cells. Additionally, an enhanced systemic response was observed, reflected in a higher percentage of CD8^+^ cells among restimulated splenocytes and a degranulation marker on all tested subpopulations of these cells.

Based on the results presented herein, as well as their comparison with those obtained previously for the treatment of MC38 cancer (26), we are convinced of the need to carefully consider the combination of individual components of therapy and to rationally analyze the emerging effects.

## Data Availability

The original contributions presented in the study are included in the article/**Supplementary Material**. Further inquiries can be directed to the corresponding author.
