# Supplementary material for: Co-delivery of IL-12/IL-15/IL-18 engineered DC vaccines with anti-IL-10R and nanoconjugated methotrexate in melanoma
Source: Front Immunol. 2026 Mar 2;17:1773836. doi: 10.3389/fimmu.2026.1773836 (PMC12989403; doi:10.3389/fimmu.2026.1773836)
Supplement: Supplementary file 1 [file DataSheet1.pdf]

Table 1 P-values for the tumor microenvironment analyses after immunotherapy determining the normality of the data distribution calculated using the Shapiro-Wilk test

	nt	Ab	I	II	III	IV	V	VI	VII	VIII	IX
CD45	0.1211	0.0460	0.0618	0.0239	0.0512	0.6830	0.2162	0.0233	0.1767	N too small	0.4243
CD8	0.4832	0.0774	0.1917	0.7166	0.5754	0.9972	0.0957	0.8205	0.7898	0.5189	0.4326
CD4	0.2868	0.2800	0.5542	0.8920	0.3710	0.2731	0.1544	0.8492	0.0033	0.1572	0.8362
Treg	0.7316	0.7342	0.4345		0.8103	0.4149	0.6563	0.6155	0.6912	0.9521	0.9556
NK	0.1010	0.4633	0.6369	0.5774	>0.9999	0.0078	0.7346	0.7935	0.5279	N too small	0.1935
TAM	0.6952	0.4402	0.3218	0.7958	0.2228	0.3248	0.4086	0.3214	0.7248	0.4813	0.5562
TAM MHC II^{high}/MHC II^{low}	0.0324	0.8482	0.4771	0.6937	0.3773	0.9121	0.1067	0.3208	0.0327	N too small	0.6777
MDSC	0.5420	0.1757	0.3214	0.7160	0.9200	0.9268	0.3032	0.3393	0.4240	0.5428	0.1268

Table 2 P-values for the tumor microenvironment analyses after chemoimmunotherapy determining the normality of the data distribution calculated using the Shapiro-Wilk test

	nt	H-M	Ab	I	II	III	IV	V	VI	VII	VIII	IX
CD45	0.0778	0.0060	0.0422	0.0040	0.2247	0.8490	0.2010	0.8043	0.2210	0.0327	0.1931	0.1489
CD8	0.1664	0.1179	0.1758	0.7862	0.4139	0.3090	0.0997	0.1013	0.5742	0.3241	0.0276	0.1664
CD4	0.2554	0.9715	0.2363	0.1923	0.5036	0.4960	0.2411	0.1219	0.5309	0.1064	0.6200	0.2376
Treg	0.8543	0.9641	0.8651	0.0523	0.9041	0.8704	0.8984	0.1252	0.6086	0.6656	0.5801	0.5637
NK	0.0454	0.3648	0.6516	0.8776	0.7571	0.4846	0.9649	0.1888	0.3180	0.7982	0.6280	0.7355
TAM	0.8967	0.4370	0.8712	0.3599	0.2560	0.4162	0.4049	0.1264	0.2748	0.2625	0.9273	0.2034
TAM MHC II^{high}/MHC II^{low}	0.0992	0.0021	0.3247	0.1956	0.4518	0.7767	0.0399	0.1449	0.6523	0.5175	0.0041	0.0365
MDSC	0.1785	0.7130	0.7326	0.8509	0.7477	0.7461	0.5153	0.9141	0.3454	0.3538	0.9846	0.2131

Table 3 P-values for the restimulated splenocytes analyses after immunotherapy determining the normality of the data distribution calculated using the Shapiro-Wilk test

	nt	Ab	I	II	III	IV	V	VI	VII	VIII	IX
CD8$^+$	0.6800	0.3650	0.7090	0.0965	0.8878	0.6516	0.5045	0.2113	0.6676	0.2282	0.3434
CD4$^+$	0.5539	0.4497	0.2177	0.3546	0.5940	0.4524	0.1437	0.5779	0.3489	0.1305	0.6849
NK	0.5293	0.6252	0.5329	0.5543	0.7189	0.6184	0.3092	0.3902	0.4080	0.2557	0.9600
CD8$^+$CD107a$^+$	0.5643	0.8619	0.0054	0.3057	0.3817	0.7042	0.0265	0.0427	0.2150	0.3815	0.5550
CD4$^+$CD107a$^+$	0.3104	0.1731	0.4068	0.4707	0.3633	0.1844	0.1573	0.4138	0.4005	0.1220	0.1065
NK CD107a$^+$	0.3921	0.1522	0.6557	0.1407	0.2262	0.7354	0.0660	0.1600	0.2185	0.4131	0.2370
IFN-γ	0.0660	0.3126	0.1916	0.2333	0.2414	0.1075	0.0267	0.4344	0.6410	0.4550	0.4327
IL-10	0.4965	0.4315	0.2138	0.1173	0.0783	0.0696	0.7688	0.2429	0.4749	0.1341	0.3292
IL-4		0.1459	0.1381	0.0708	0.6949	0.0783	0.1701	0.0919	0.4031	0.5941	0.2968

Table 4 P-values for the restimulated splenocytes analyses after chemoimmunotherapy determining the normality of the data distribution calculated using the Shapiro-Wilk test

	nt	H-M	Ab	I	II	III	IV	V	VI	VII	VIII	IX
CD8$^+$	0.6027	0.8095	0.8673	0.3677	0.3184	0.8107	0.2632	0.0191	0.0952	0.6294	0.7570	0.4769
CD4$^+$	0.1650	0.3905	0.5334	0.5481	0.3485	0.9386	0.6411	0.4817	0.3553	0.1639	0.7256	0.0900
NK	0.3909	0.6103	0.4647	0.2243	0.5455	0.0555	0.7968	0.1818	0.9775	0.0512	0.8644	0.2579
CD8$^+$CD107a$^+$	0.7839	0.6304	0.2995	0.8103	0.0270	0.7098	0.8564	0.3064	0.3656	0.8925	0.5381	0.7237
CD4$^+$CD107a$^+$	0.3264	0.9649	0.3174	0.7833	0.1869	0.9104	0.2012	0.6214	0.1564	0.2626	0.7036	0.4030
NK CD107a$^+$	0.5775	0.7352	0.1618	0.8968	0.4654	0.3978	0.8881	0.2311	0.7903	0.2213	0.1391	0.2121
IFN-γ	0.9734	0.6950	0.2524	0.2832	0.1750	0.9764	0.1427	0.8598	0.2587	0.8344	0.3263	0.2980
IL-10	0.5952	0.5139	0.1045	0.4305	0.3363	0.6350	0.7504	0.1317	0.1187	0.2791	0.7103	0.2160
IL-4			<0.0001	0.9417	0.0691	0.4928	0.4921	0.9445	0.4857	0.1743	0.8451	0.0365
